# Coplanar Floating-Gate Antiferroelectric Transistor with Multifunctionality for All-in-One Analog Reservoir Computing

**DOI:** 10.1007/s40820-025-02049-9

**Published:** 2026-01-08

**Authors:** Yufei Shi, Zijie Zheng, Jiali Huo, Yu-Chieh Chien, Sifan Li, Haofei Zheng, Xiao Gong, Kah-Wee Ang

**Affiliations:** Department of Electrical and Computer Engineering, National University of Singapore, 4 Engineering Drive 3, Singapore, 117583 Singapore

**Keywords:** Hafnium zirconium oxide, Coplanar structure, Antiferroelectric field-effect transistor, Reservoir computing, Multifunctionality

## Abstract

Analog reservoir computing (ARC) systems offer an energy-efficient platform for temporal information processing. However, their physical implementation typically requires disparate materials and device architectures for different system components, leading to complicated fabrication processes and increased system complexity. In this work, we present a coplanar floating-gate antiferroelectric field-effect transistor (FG AFeFET) that unifies multiple neural functionalities within a single device, enabling the physical implementation of a complete ARC system. By combining a coplanar layout design with an area ratio engineering strategy, we achieve tunable device behaviors, including volatile responses for artificial neuron emulation, nonvolatile states for synaptic functions, and fading memory dynamics for reservoir operations. The mechanisms underlying these functionalities and their operating mechanism are systematically elucidated using load line analysis and energy band diagrams. Leveraging these insights, we demonstrate an all-in-one ARC system based on the unified coplanar FG AFeFET architecture, which achieves recognition accuracies of 95.6% and 83.4% on the MNIST and Fashion-MNIST datasets, respectively. These findings highlight the potential of coplanar FG AFeFETs to deliver area-efficient, design-flexible neuromorphic hardware for next-generation computing systems.

**Supplementary Information:**

The online version contains supplementary material available at 10.1007/s40820-025-02049-9.

## Highlights

- A novel coplanar structure design is proposed for floating-gate antiferroelectric field-effect transistor (FG AFeFET) demonstration with enhanced design flexibility and vertical scalability.
- Multifunctionality is achieved within a single coplanar FG AFeFET via area ratio engineering, including volatile neuronal behavior, fading memory dynamics, and nonvolatile synaptic function. Systematic investigations into its detailed operating principles are conducted.
- Seamless integration of a full analog reservoir computing system is demonstrated based on a unified coplanar FG AFeFET architecture, realizing satisfactory accuracies for pattern recognition tasks.

**Supplementary Information:**

The online version contains supplementary material available at 10.1007/s40820-025-02049-9.

## Graphical abstract

**Figure:**
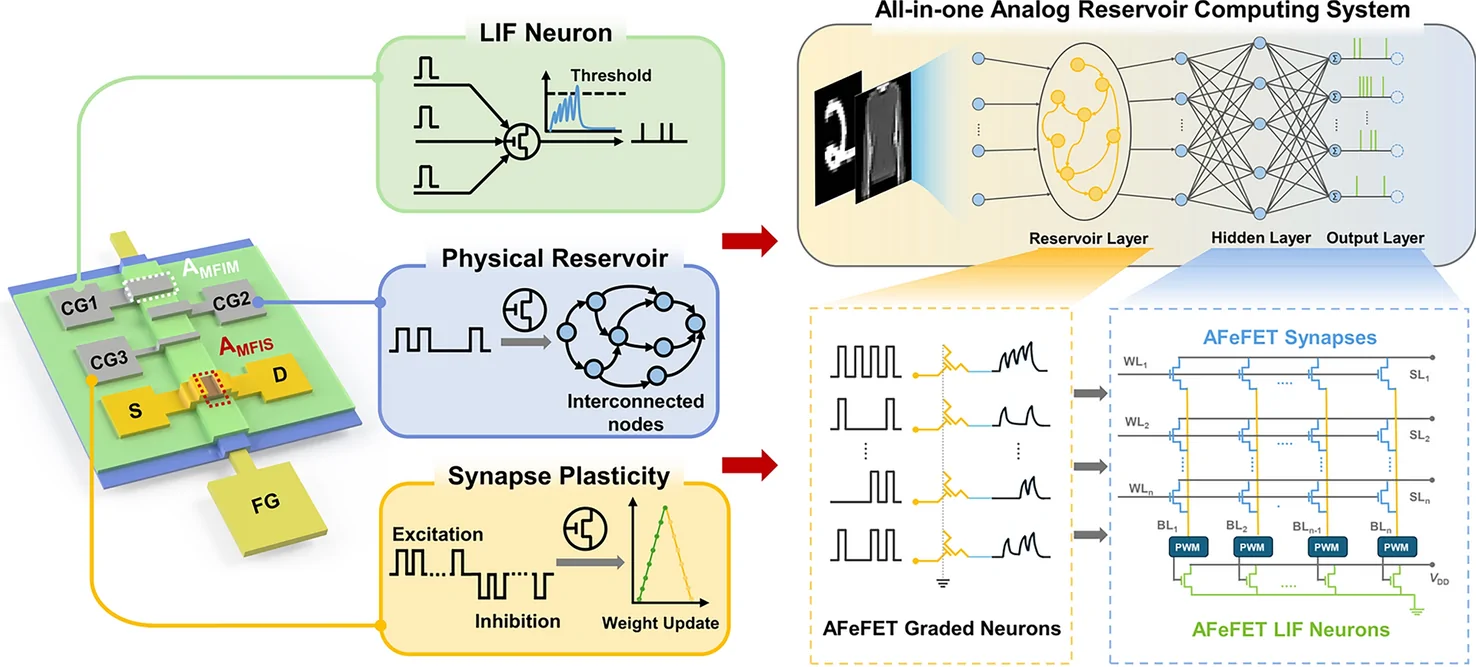

## Introduction

The growing demand for efficient processing of data-intensive and spatiotemporal tasks has spurred interest in specialized hardware beyond conventional computing paradigms [1, 2]. Inspired by biological neural networks, neuromorphic computing provides a promising approach to emulate the brain’s information processing through hardware capable of supporting diverse functionalities. Within this paradigm, analog reservoir computing (ARC) has emerged as a powerful framework for temporal information processing [3]. A typical ARC system comprises a dynamic reservoir that transforms complex input signals into a high-dimensional state space, thereby enhancing feature differentiation, together with a trainable readout layer that performs post-processing of the resulting reservoir state. A key requirement for hardware implementation of such systems is the development of compact, energy-efficient, and scalable devices capable of emulating diverse neural behaviors, including both volatile nonlinear dynamics and nonvolatile characteristics within a unified architecture [4–7]. In this context, devices with tunable characteristics or multifunctionality are gaining attention for their ability to leverage the analog and nonlinear characteristics of specific materials and architectures. However, the conventional complementary metal–oxide–semiconductor (CMOS) technology-based ARC system suffers from limited density and energy efficiency because it necessitates complex circuit design to mimic nonlinear dynamics and neural operations [8–10]. Despite emerging devices such as memristors offering alternatives due to their inherent biological resemblance [11–15], current physical implementation of ARC systems still relies on distinct materials or device structures and requires separate fabrication processes, which increases fabrication complexity and hinders seamless integration of the overall system [16].

Hafnium zirconium oxide (HZO) has recently gained attention owing to its reliable polarization switching characteristic, tunability via material composition engineering, and the capability of maintaining ferroelectric and antiferroelectric properties at nanoscale thicknesses [17, 18]. However, ferroelectric Hf_0.5_Zr_0.5_O_2_ is widely used for nonvolatile memories and presents intrinsic limitations for implementing neuron-like transient behavior without additional feedback circuitry, which increases energy consumption and circuit complexity [19, 20]. In contrast, antiferroelectric Hf_0.25_Zr_0.75_O_2_ offers spontaneous depolarization upon field removal, making it inherently suitable for nonlinear dynamics and volatile behavior emulation [21–23]. And its incorporation with a floating-gate (FG) device structure further enables the realization of nonvolatility [24–26]. Despite both volatile and nonvolatile characteristics having been demonstrated in HZO-based FETs, their unified implementation within a single device remains unachieved. Additionally, maintaining nonvolatility in floating-gate antiferroelectric field-effect transistor (FG AFeFET) often requires constant read bias, leading to increased standby energy consumption. Besides, existing studies primarily focused on the static performance of the FG AFeFET, with limited understanding of the underlying mechanisms related to its dynamic behavior.

**Fig. 1:**
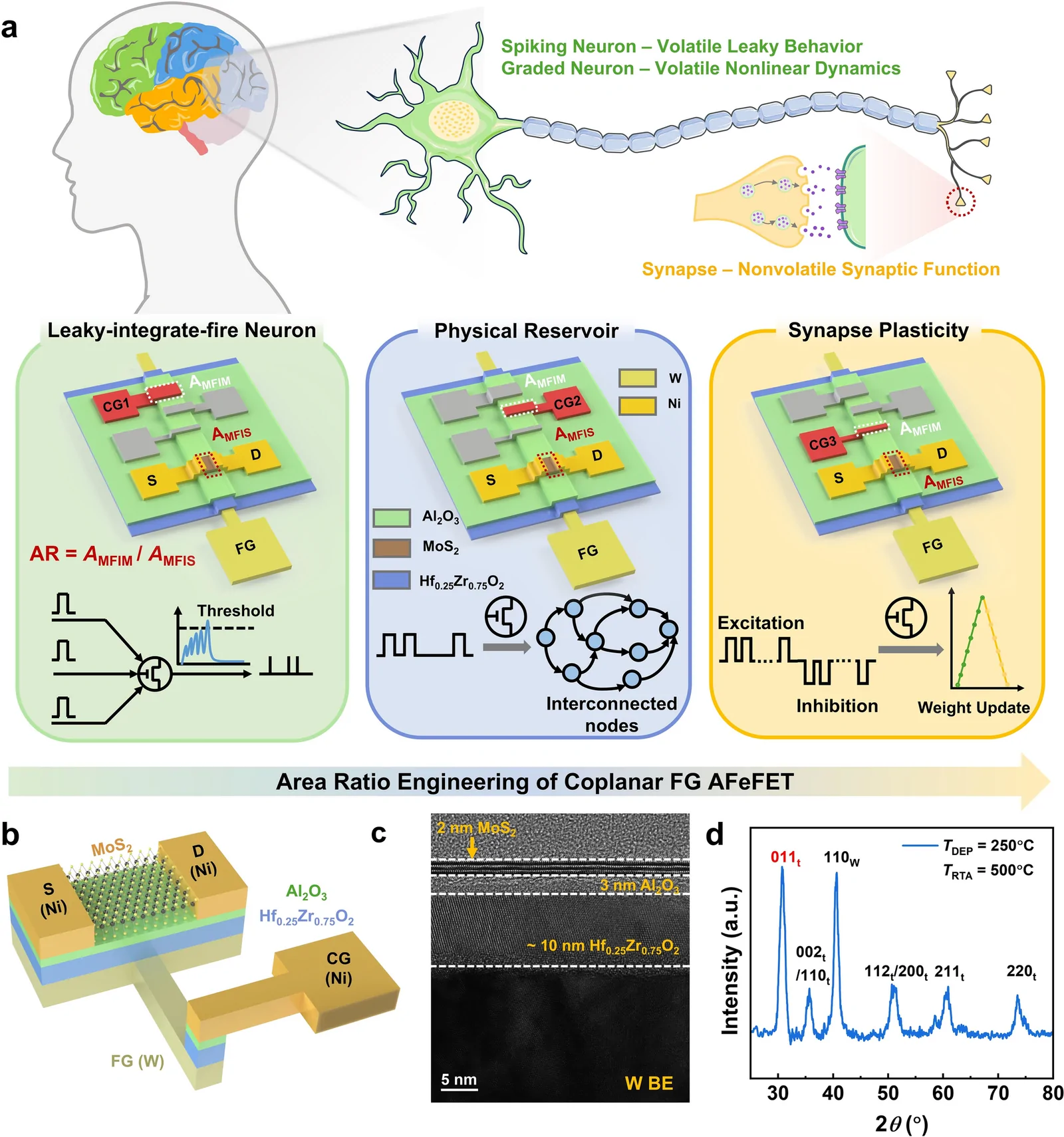
Coplanar floating-gate AFeFET with tunable area ratio for biological system emulation. **a** Schematic demonstration of the projection between biological neural functionalities and tunable electrical behaviors of coplanar FG AFeFET. The 3D schematic illustrates the device structure of the coplanar FG AFeFET, featuring a tunable area ratio. **b** Cross-sectional view of the coplanar FG AFeFET, further clarifying the proposed coplanar FG architecture with the control-gate last fabrication process. **c** Cross-sectional TEM images of the gate stack for the fabricated coplanar FG AFeFET. **d** Grazing incidence X-ray diffraction (GIXRD) spectrum of the Hf_0.25_Zr_0.75_O_2_ layer, with 2*θ* ranging from 25° to 80°. The presence of the (011)_t_ diffraction peak at 2*θ* ≈ 30.7° indicates the existence of the antiferroelectric tetragonal phase

In this work, we introduce a coplanar floating-gate AFeFET through device configuration engineering to realize ARC system implementation within a unified device platform (Fig. 1a). The incorporation of a multigate coplanar FG architecture allows flexible modulation of device behavior through area ratio (AR) engineering, enabling three distinct operational modes: leaky–integrate–fire (LIF) neuron behavior, physical reservoir dynamics, and synaptic plasticity. This unified approach integrates multiple neural functionalities within a single device, eliminating the need for heterogeneous device types or separate fabrication processes. Besides, the proposed control-gate-last fabrication process for coplanar FG AFeFET enables floating-gate functionality through a metal–antiferroelectric–insulator–semiconductor (MFIS) stack without requiring complex additional steps. This approach not only simplifies the fabrication process but also enhances the vertical scalability compared to conventional FG AFeFETs. Using this architecture, we demonstrate a coplanar FG AFeFET-based ARC system, achieving recognition accuracies of 95.6% and 83.4% on MNIST and Fashion-MNIST datasets, respectively. These results underscore the potential of coplanar FG AFeFET for compact and energy-efficient neuromorphic hardware with high functional integration.

## Experimental Section

### Device Fabrication

A 30 nm tungsten (W) layer was first sputtered on a p-type silicon substrate with 285 nm SiO_2_ and followed by a lithography process to form the pattern of the back electrode, which is used as the floating gate. Then the area uncovered by the photoresist is etched using tungsten etchant with DI water rinsing. Then 10 nm Hf_0.25_Zr_0.75_O_2_ film was deposited using the thermal atomic layer deposition (ALD) at 250 °C. The Tetrakis (ethylmethylamido) hafnium, Hf[N-(C_2_H_5_)CH_3_]_4_ and Tetrakis (ethylmethylamino) zirconium, Zr[N-(C_2_H_5_)CH_3_]_4_, and ozone were used as precursors during the deposition process. Following this, 30 nm W layer was sputtered on top of the Hf_0.25_Zr_0.75_O_2_ before subjecting the device to the rapid thermal annealing process at 500 °C for 60 s. The sacrificial W layer was removed by tungsten etchant after the annealing, and a 3 nm Al_2_O_3_ layer was deposited with ALD at 150 °C as the insulating layer. Next, exfoliated few-layer MoS_2_ flakes were transferred, serving as the semiconducting channel. Finally, the patterns of source/drain contacts together with the top gate electrodes (control gates) were defined by the electron beam lithography, followed by 25 nm Ni evaporated on top using the electron beam evaporator and a lift-off process. The Ni deposition rate is optimally selected at 0.3 Å s^−1^ to minimize the bombardment damage on the MoS_2_ top surface.

### Characterization and Measurement

The electrical characterizations, including DC *I*-*V* measurements and transient pulse *I*-*V* measurements, were done by the Keysight B1500A semiconductor analyzer under the dark ambient environment at room temperature. The Keysight B1530 with a waveform generator fast measurement unit (WGFMU) was used to generate the designed waveforms for pulse *I*-*V* measurements. In addition, the antiferroelectric characteristics related to the MFM and MFIM capacitors were measured using an aixACCT TF 3000 analyzer. Cross-sectional transmission electron microscopy (TEM) and energy-dispersive X-ray spectroscopy (EDS) mapping were conducted using a Talos F200X TEM to confirm the device structure of the fabricated coplanar FG AFeFET. And grazing incidence X-ray diffraction using Cu *k*α radiation is utilized to characterize the crystalline phase of the deposited antiferroelectric Hf_0.25_Zr_0.75_O_2_ film.

## Results and Discussion

### Area Ratio Engineering of Coplanar FG AFeFET with Multigate Design

The device structure of the fabricated FG AFeFET is illustrated in Fig. 1a, b. To enhance vertical scalability by reducing the required thickness of the HZO layer *t*_HZO_ while preserving the tunability of the floating-gate architecture, a coplanar structure design employing a control-gate-last fabrication process is adopted. Unlike the conventional FG structure, where the bottom electrode serves as the control gate (CG), the coplanar FG AFeFET utilizes the bottom electrode as the floating gate. The control gate is formed on the top surface in the final fabrication step, alongside the source and drain formation. This configuration decouples the device into the channel region (metal–antiferroelectric–insulator–semiconductor: MFIS) and the AFE region (metal–antiferroelectric–insulator–metal: MFIM). The area of these two regions is denoted as *A*_MFIS_ and *A*_MFIM_ and determined by the FG/CG overlap (labeled with the white rectangle in Fig. 1a) and FG/channel overlap (red rectangle), respectively. These regions form two capacitive elements, *C*_MFIS_ and *C*_MFIM_, and make the voltage division across the device governed by the designed area ratio *A*_MFIM_/*A*_MFIS_, as described in Eq. 1 and detailed in Fig. S1 and Note S1.

1 $$\frac{{V_{{{\mathrm{MFIM}}}} }}{{V_{{{\mathrm{MFIS}}}} }} = \frac{{A_{{{\mathrm{MFIS}}}} }}{{A_{{{\mathrm{MFIM}}}} }} \cdot \frac{{\frac{{d_{{{\mathrm{HZO}}}} }}{{\varepsilon_{{{\mathrm{HZO}}}} }} + \frac{{d_{{{\mathrm{ins}}}} }}{{\varepsilon_{{{\mathrm{ins}}}} }}}}{{\frac{{d_{{{\mathrm{HZO}}}} }}{{\varepsilon_{{{\mathrm{HZO}}}} }} + \frac{{d_{{{\mathrm{ins}}}} }}{{\varepsilon_{{{\mathrm{ins}}}} }} + \frac{{d_{{\mathrm{S}}} }}{{\varepsilon_{{\mathrm{S}}} }}}} \approx \frac{{A_{{{\mathrm{MFIS}}}} }}{{A_{{{\mathrm{MFIM}}}} }}$$

This straightforward inverse relationship between the voltage distribution and designed area ratio outperforms conventional FG FE/AFeFETs, where the voltage division is more complex, and the area ratio is fixed once the channel is formed. Such constraints limit the range of achievable ARs in conventional FG FE/AFeFETs, especially in devices with two-dimensional (2D) material channels, which often exhibit irregular shapes and limited size. Differently, in the coplanar structure, *A*_MFIM_ can be defined after channel formation, offering greater flexibility in AR design. This enables the fabrication of multiple control gates to form varying *A*_MFIM_ within a single device, facilitating the systematic investigation of AR-dependent device behavior. Moreover, the floating-gate-like structure is successfully incorporated into the coplanar FG AFeFET with a simplified MFIS fabrication process, requiring only a 10 nm thin Hf_0.25_Zr_0.75_O_2_ layer. As shown in Fig. 1c, the multilayer gate stacks of the fabricated device were characterized using cross-sectional transmission electron microscopy (TEM), revealing sharp and well-defined interfaces between the different layers. Energy-dispersive spectrometry (EDS) shown in Fig. S2 confirms a uniform elemental distribution across the device, with no detectable interdiffusion between layers. A 2D MoS_2_ channel was utilized due to its high mobility at atomic thickness and superior gate control capability in thin-film devices [27–30]. Additionally, peaks at 2*θ* ≈ 30.7° shown in grazing incidence X-ray diffraction (GIXRD) confirm the presence of the antiferroelectric tetragonal phase in the deposited Hf_0.25_Zr_0.75_O_2_ thin film (Fig. 1d).

**Fig. 2:**
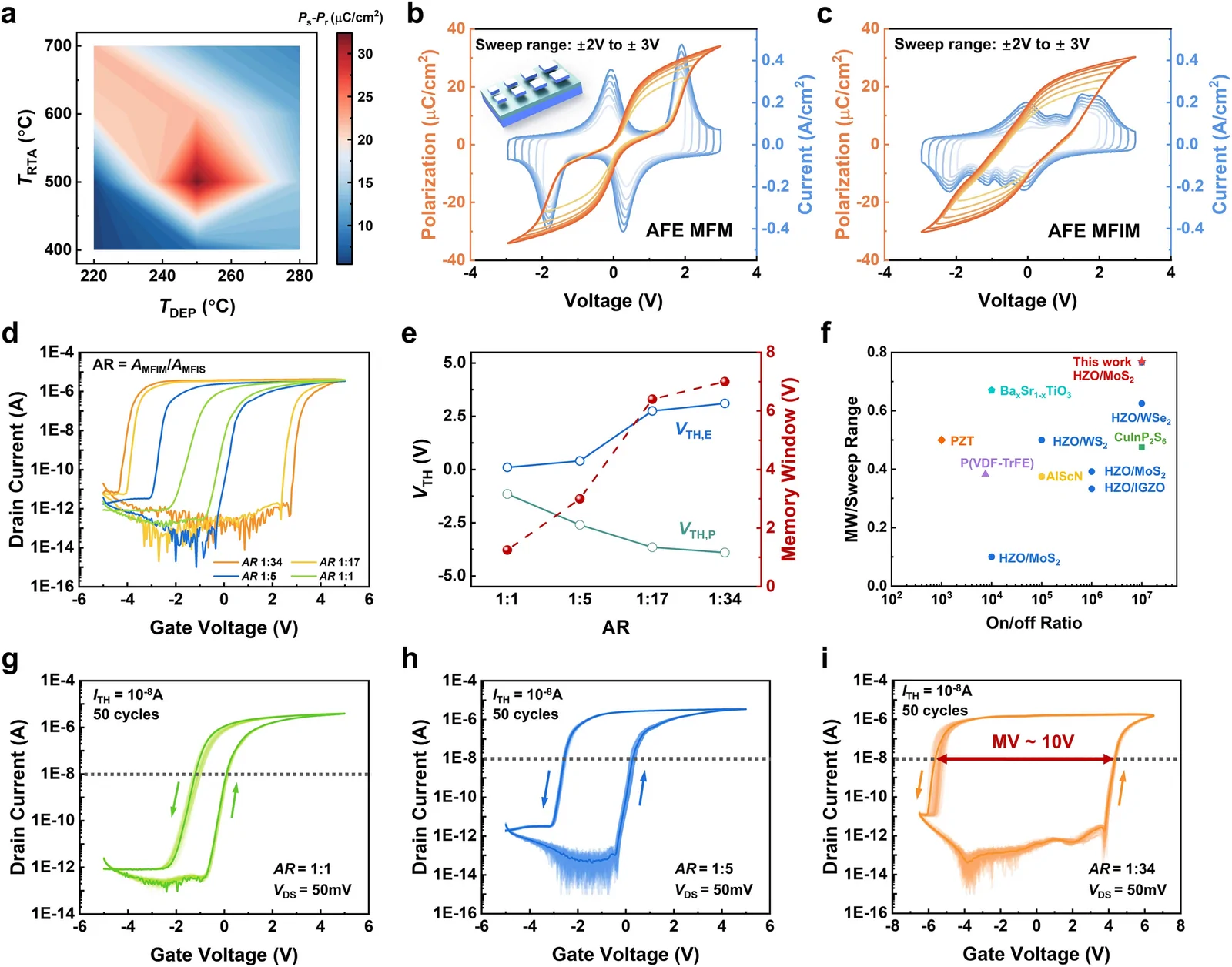
Device performance of coplanar FG AFeFET. **a** Evolution of (*P*_s_−*P*_r_) value under different fabrication conditions. **b** Polarization and displacement current of the MFM capacitor as a function of applied voltages. Typical double hysteresis curves with partial polarization effect under low sweeping voltages are obtained. **c** *P*–*V* and *I*-*V* curves of the MFIM capacitor, showing a transition toward FE-like behavior. **d** Variation of transfer characteristics with different area ratios, revealing the effective tunability of area ratio on the memory window. **e** Evolution of *V*_TH_ for both program state *V*_TH,P_ and erase state *V*_TH,E_, and the obtained memory window as a function of area ratio. **f** Benchmark of memory window efficiency and on/off ratio of the coplanar FG AFeFET compared to previously reported ferroelectric devices. Transfer characteristic of the device with **g** AR = 1:1. **h** AR = 1:5. **i** AR = 1:34. Stable counterclockwise hysteresis with a high on/off ratio is well-sustained under 50 consecutive dual-sweeping cycles

In Fig. 2a, polarization data extracted from metal–antiferroelectric–metal (MFM) capacitors fabricated at various deposition temperatures *T*_DEP_ and rapid thermal annealing temperatures *T*_RTA_ show that the highest polarization difference between saturation and remanent polarization (*P*_s_-*P*_r_) is achieved at *T*_DEP_ = 250 °C with *T*_RTA_ = 500 °C, indicating a promising AFE characteristic. This fabrication condition is adopted in the following discussion. The corresponding polarization–voltage (*P*–*V*) hysteresis, displacement current–voltage (*I*–*V*) loops, and capacitance–voltage (*C–V*) curves of the deposited Hf_0.25_Zr_0.75_O_2_ film are shown in Figs. 2b and S3a, which exhibit a representative double hysteresis in the measured *P*–*V* curve and a double-humped shape with four capacitance peaks in the *C*-*V* curve. The obtained AFE behavior can sustain over 10^7^ cycles of repeated stressing pulses (Fig. S4). To accurately capture the antiferroelectric behavior of the deposited Hf_0.25_Zr_0.75_O_2_ film in the designed gate stack, an MFIM capacitor is further fabricated for characterization. Compared to the representative AFE double hysteresis *P*–*V* loop in the MFM capacitor, the insertion of a 3 nm thin Al_2_O_3_ layer results in a shift toward ferroelectric-like (FE-like) behavior, as evidenced by a single hysteresis loop and significantly increased *P*_r_, as well as butterfly-shaped capacitance curves (Fig. S3b). This interlayer-caused *P*–*V* hysteresis shift together with the symmetric gate stack design results in distinct operating principles within the device compared to conventional FG AFeFET, which will be discussed in detail in the following sections.

Following the characterization of antiferroelectricity in the AFE gate stack, the transfer characteristics of the fabricated FG AFeFET were evaluated across area ratios ranging from 1:34 to 1:1 under a ± 5 V gate voltage *V*_GS_ applied to different control gates (Fig. 2d). All configurations exhibit counterclockwise hysteresis, and the corresponding memory window (MW) extracted from the transfer curves shows a monotonic increase as AR decreases (Fig. 2e). Ultimately, a large memory window of around 10 V is achieved at a small AR of 1:34 (under a ± 6.5 V applied *V*_GS_) with a high on/off ratio > 10^7^ obtained at the same time, exhibiting overall improvement compared to previously reported ferroelectric devices (Fig. 2f) [31–41]. In Fig. 2g–i, cycling tests for three representative AR conditions are conducted, including AR = 1:1 (MFIS and MFIM balanced condition), AR = 1:5 (intermediate condition), and AR = 1:34 (MFIM-dominated condition). The obtained results show stable transfer characteristics across 50 cycles, with minimal cycle-to-cycle variation and a well-sustained high on/off ratio. Besides the AR-dependent memory window expansion, the coplanar FG AFeFET also exhibits an AR engineering-enabled tunable memory behavior. Detailed characterization of the device’s memory behavior under different ARs and corresponding operating principles is provided in the following discussion.

### Volatile FG AFeFET for LIF Neuron

**Fig. 3:**
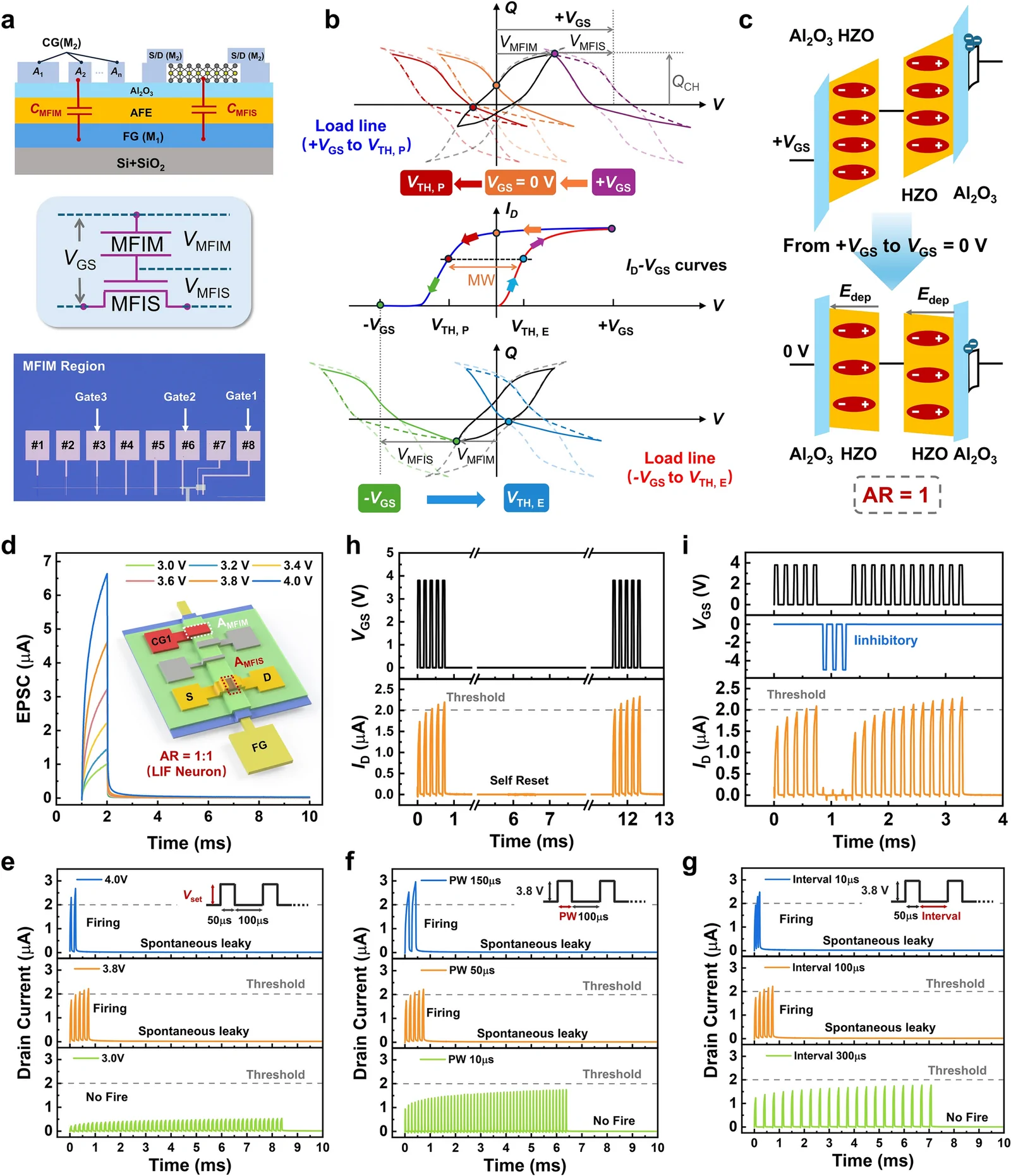
LIF characteristic of coplanar FG AFeFET with a large AR. **a** Schematic of the equivalent capacitance model and optical microscopy image of the control gate design. **b** Load line analysis of coplanar FG AFeFET with AR = 1:1. Upper panel: processes from applying + *V*_GS_ to *V*_GS_ = 0 V to *V*_GS_ = *V*_TH, P_; bottom panel: process from applying -*V*_GS_ to *V*_GS_ = *V*_TH, E_. Middle panel: theoretical *I*_D_−*V*_GS_ curve obtained from load line analysis. **c** Band diagram of the gate stack corresponds to processes from applying + *V*_GS_ to *V*_GS_ = 0 V. It is consistent with the load line analysis, while the *P*_r_ of HZO can retain the carriers in the channel after removing + *V*_GS_. However, poor retention could be expected due to the depolarization field and the limited *E*_c_ of HZO layer. **d** Drain current integration and leaky process of the FG AFeFET under the stimulation of a single positive pulse ranging from 3.0 V to 4.0 V, showing volatile behavior with fast current decay. The inset schematic demonstration shows the activation of CG1 under a large AR configuration. Demonstration of neuronal behavior modulation through **e** different applied voltage amplitudes (fixed 50 µs pulse width and 100 µs pulse interval), **f** different pulse widths (fixed 3.8 V pulse amplitude and 100 µs pulse interval), and **g** different pulse intervals (fixed 3.8 V pulse amplitude and 50 µs pulse width). Higher stimulation intensity accelerates the arrival of firing events, while insufficient stimulation is unable to activate the firing events. **h** Device response to two successive groups of stimulation pulses, showing self-reset characteristic during the voltage removal and reactivation of LIF behavior after the reset. **i** Response of the device to inhibitory stimuli

Taking advantage of the coplanar device structure, various area ratios are achieved by designing multiple FG/CG overlaps to create different *A*_MFIM_ while keeping the *A*_MFIS_ unchanged. The optical microscopy image of the control gate design is shown in Fig. 3a, and detailed geometric parameters are provided in Fig. S5 and Tables S1 and S2. During characterization of the AR engineering effect in the multigate structure, the large AR of 1:1 functions as a transition point for the device to operate from an MFIS-dominated regime (large AR ≥ 1) to an MFIM-dominated regime (AR < 1), during which the majority of the applied voltage drop gradually shifts from the MFIS region to the MFIM region. To carefully investigate the device performance under this specific AR, we model the device as serial-connected MFIM and MFIS stacks (Fig. 3a). Through load line analysis combined with the corresponding energy band diagram, its operating principle is systematically investigated (Fig. 3b, c). A detailed description of analytical procedures and the basis of the load line method are provided in Note S2 and Fig. S6 [42]. In addition, the load line graph for the device operating under a fixed + *V*_GS_ is exhibited in Fig. S7 as an example to explain the analysis process.

In Fig. 3b, the upper panel depicts the analysis process when *V*_GS_ sweeps from + *V*_GS_ to 0 V and then back to *V*_TH,P_. For the device in the on-state, the voltage drop across the channel layer is negligible, thereby the *Q*–*V* relations of the gate stacks on both sides of the FG are nearly identical, leading to an equal voltage division. When + *V*_GS_ is applied, the HZO layers on both sides of the FG reach the same polarization state, and an operating point with a high *Q* value can be obtained from the load line graph (labeled using a purple dot in the upper panel). This high total charge indicates carrier accumulation in the channel layer and results in a large drain current *I*_D_, as labeled by the purple dot on the *I*_D_–*V*_GS_ curve in the middle panel. When *V*_GS_ returns to 0 V, the *P*_r_ of the HZO layer preserves the channel carriers, maintaining the on-state (orange operation point). The corresponding band diagram evolution from the purple to orange operation points is illustrated in Fig. 3c. However, the depolarization field (*E*_dep_) and finite coercive field (*E*_c_) inevitably degrade polarization stability, leading to limited retention. (A detailed investigation of back-switching characteristic in intrinsic AFE film is provided in Fig. S8 and Note S3.) Finally, the red load line in the upper panel of Fig. 3b and its associated operation point represent the transition of the channel into depletion. Beyond this stage, the finite dielectric constant and voltage drop in the channel layer can no longer be neglected; the effective area of the MFIS region decreases, synergistically compressing the *Q*–*V* curve of the MFIS part. Consequently, in the bottom panel of load line analysis for applying − *V*_GS_, the HZO layer in the MFIM region cannot achieve saturated switching. This leads to asymmetric threshold voltages *V*_TH,E_ and *V*_TH,P_, with *V*_TH,E_ shifted closer to 0 V. Details related to different operating points in Fig. 3b are illustrated in Fig. S9. These operating points obtained via load line analysis constitute an analytical *I*_D_-*V*_GS_ curve, as shown in the middle panel of Fig. 3b. The obtained curve through this analysis shows strong agreement with the measured *I*_D_–*V*_GS_ characteristics (Fig. 2g), offering a qualitative explanation of the experimental results.

After the theoretical analysis, the volatile response is verified through excitatory-post-synaptic-current (EPSC) measurements (Fig. 3d). Upon the application of an excitatory pulse, progressive polarization switching in the AFE layer results in obvious current integration, with increasing pulse widths yielding stronger responses. The subsequent rapid decay in *I*_D_ upon voltage removal confirms the volatility of the device. The observed volatile behavior is further validated by EPSC measurement on 10 devices, all of which show a consistent fast decay with small device-to-device (D2D) variation (Fig. S10). This current integration and leaky behavior preserve over a wide range of gate voltages, enabling self-reset functionality analogous to biological neurons and obviating the need for external reset circuitry. Figure 3e presents the device response to excitatory pulses, which exhibits gradual current integration during pulses and spontaneous decay in between. Upon reaching a threshold value (*I*_TH_ = 2 µA), a firing event occurs, followed by a spontaneous reset to its initial state. Notably, this LIF behavior is sensitive to pulse parameters. Increasing pulse amplitude (Fig. 3e) or width (Fig. 3f) reduces the number of pulses required for firing, which can be attributed to enhanced tetragonal-to-orthorhombic phase transitions in the AFE layer under stronger fields [43]. The resulting increase in polarized dipoles modulates channel conductance more effectively, accelerating current integration. The pulse interval also plays a critical role. Longer intervals exacerbate depolarization-driven decay, requiring more pulses to reach the threshold (Fig. 3g). However, if pulse intensity is insufficient due to low amplitude, short duration, or long intervals, the device fails to fire. The suppression of firing behavior occurs because the weak current integration fails to compensate for the leaky component, or the pronounced leakage effect surpasses the accumulation of polarized charges. This characteristic further demonstrates the filtering function of the AFeFET neuron. The reproducibility of LIF behavior is demonstrated in Fig. 3h, where the device successfully resets and fires under two successive groups of excitatory pulses. Furthermore, inhibitory pulse responses are illustrated in Fig. 3i. The insertion of inhibitory pulses between excitatory pulse trains effectively suppresses current integration and resets the neuron, highlighting the AFeFET's bidirectional response capability.

### Nonvolatile FG AFeFET for Synaptic Function

**Fig. 4:**
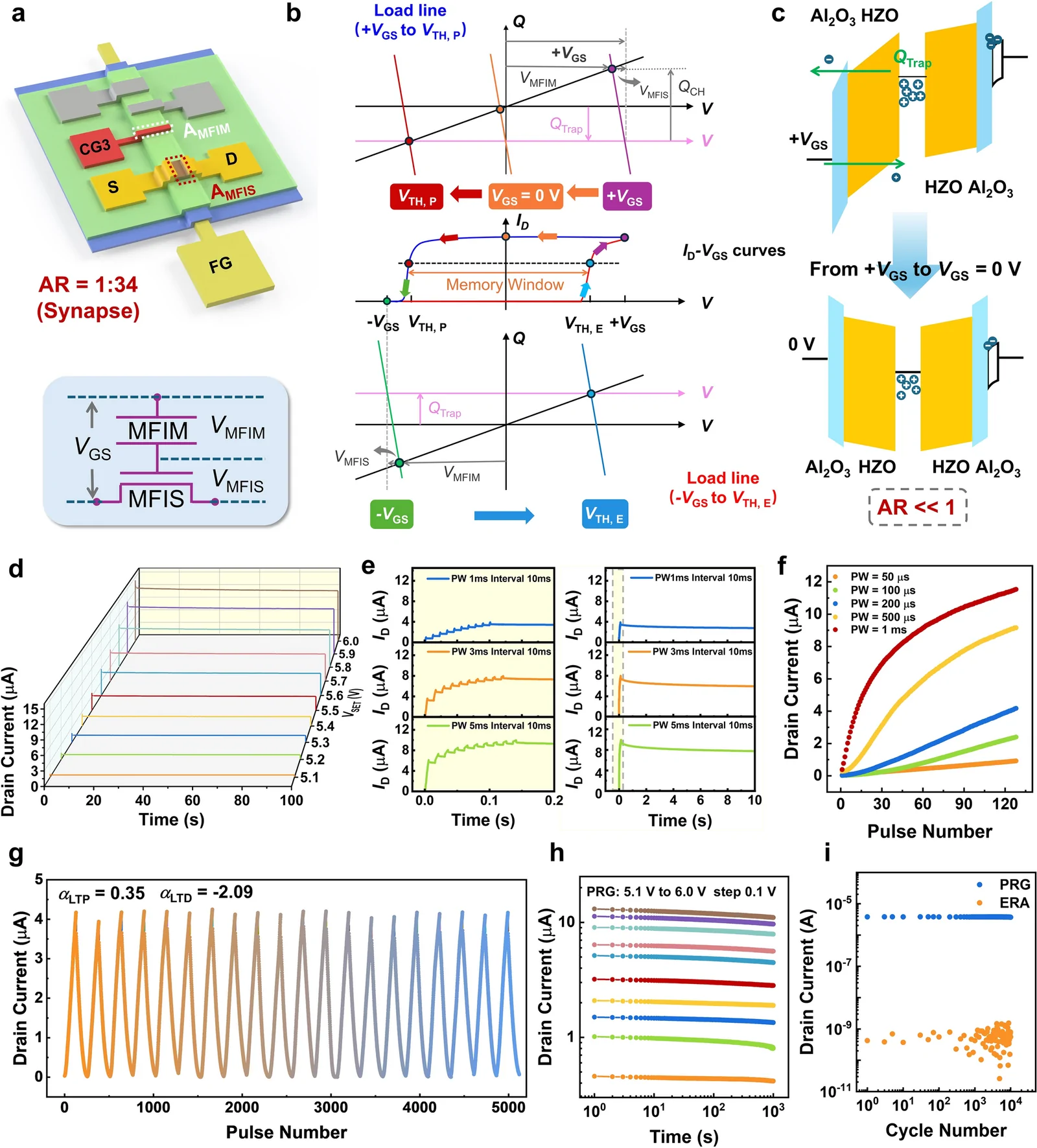
Nonvolatile characteristic of coplanar FG AFeFET with small AR for biological synapse emulation. **a** Schematic demonstration that shows activation of CG3 under the small AR configuration. **b** Load line analysis of coplanar FG AFeFET with AR ≪ 1. The *Q*–*V* curves of MFIM part and MFIS part are simplified into straight lines, due to the small *A*_MFIM_ and the extremely limited switching of the HZO layer within MFIS part, respectively. **c** Band diagram of the gate stack corresponds to the processes from applying + *V*_GS_ to *V*_GS_ = 0 V. **d** Evolution of drain current under the stimulation of a single excitatory pulse with varying pulse amplitudes (*t*_pw_ = 3 ms). **e** Modulation of current integration behavior with different pulse widths (*V*_set_ = 5.5 V). The negligible current decay during pulse interval (left panel) and maintained drain current after removing applied pulses (right panel) provide further evidence for the presence of nonvolatility. **f** Long-term potentiation characteristic of the FG AFeFET under 128 identical programming pulses when different pulse widths are adopted. **g** Cycling validation of LTP and LTD characteristics of the device with 256 pulses included in each cycle (LTP: identical pulses *V*_GS_ = 5.5 V, *t*_PW_ = 200 µs, interval = 50 µs, LTD: nonidentical pulses *V*_GS_ =  − 4.53 V to − 5.8 V with a step of − 0.01 V, *t*_PW_ = 200 µs, interval = 50 µs). **h** Retention characteristics of drain current corresponding to ten different conductance states across 1000 s. **i** Endurance characteristic of the device

In contrast to the volatile behavior obtained under a large AR of 1:1, nonvolatile characteristics become dominant when the size of *A*_MFIM_ is significantly reduced to form a small AR of 1:34 (AR ≪ 1), where *A*_MFIM_ is far smaller than *A*_MFIS_ (Fig. 4a). The corresponding load line analysis is demonstrated in Fig. 4b. In this case, the MFIS and MFIM regions experience a highly unbalanced voltage distribution. Considering that the area of the MFIS region remains unchanged in practical devices, the reduced MFIM area is reflected in the load line plot as a compression of its *Q*–*V* curve along the y-axis. For example, when AR = 1:34, the *Q* value is reduced by a factor of 34. As a result, when + *V*_GS_ is applied, a large portion of the voltage drop occurs across the MFIM region to maintain the continuity of the electric displacement. Due to the very small voltage drop across the MFIS region, the HZO layer within it hardly undergoes polarization switching. For clarity, we use straight lines to represent the *Q*–*V* relations of both MFIM and MFIS regions, with their slope ratio approximately equal to AR. This simplification does not affect the mechanism analysis.

Referring to the experimentally measured memory window, which already far exceeds the upper limit solely attributable to the ferroelectricity of HZO (2*E*_c_ × *t*_HZO_), it is evident that charge trapping dominates the device operation in this regime. Under + *V*_GS_, non-negligible positive charges are trapped within FG because *V*_MFIM_ is significantly larger than *V*_MFIS_, leading to a substantial negative shift in the *V*_TH_ of the device. Based on the load line analysis, trapped charges *Q*_Trap_ manifest as a vertical shift of the *Q*–*V* curve (refer to Note S2), as indicated by the pink x-axis in Fig. 4b. Therefore, channel charges *Q*_CH_ remain positive when *V*_GS_ sweeps from + *V*_GS_ to 0 V, keeping the device in the on-state (from the purple operation point to the orange operation point) until reaching *V*_TH,P_ (red operation point). The bottom panel of Fig. 4b shows the load line plot while applying − *V*_GS_ and *V*_TH,E_. The overall device operation mechanism is similar to that of FG memories. Given the relatively small electric field for detrapping and the effective blocking provided by the Al_2_O_3_ layer, good retention performance can be expected. A consistent process can also be obtained from the energy band diagram evolution shown in Fig. 4c. Under this small AR, the significantly enhanced voltage drop across the MFIM region (*V*_MFIM_) strengthens corresponding polarization and induces significant electron trapping along the FG to CG direction. Upon voltage removal, the positive trapped charge in the FG layer maintains the downward band bending in the channel, enabling long-term memory functionality. This nonvolatile behavior, combined with partial polarization switching, allows the device to mimic synaptic plasticity.

The nonvolatile behavior of the device is first evaluated using a read-after-write measurement (Fig. 4d). A single programming pulse induces a clear increase in *I*_D_, which remains stable for over 100 s with minimal decay. Robust nonvolatile retention is sustained across various pulse conditions, with higher programming voltages amplifying the response. In addition, the device’s multilevel memory capability is demonstrated through a sequence of programming pulses. Under ten consecutive pulses, the drain current accumulates and remains stable between pulses, verifying the device’s ability to support continuous and stable conductance updates (Fig. 4e). The varying pulse width shows a clear modulation effect on the linearity of the synaptic response. As good linearity is crucial for the synaptic device to realize reliable and stable weight updates, long-term potentiation (LTP) measurement over a wide range of pulse width *t*_pw_ is further performed to explore the influence of pulsing parameters on synaptic plasticity. As shown in Fig. 4f, multilevel synaptic weights are obtained across all *t*_pw_ conditions when a sequence of 128 pulses with identical pulsing parameters is applied to the control gate. High linearity is observed for *t*_pw_ shorter than 200 µs but begins to degrade at a *t*_pw_ of 500 µs due to enhanced dipoles switching under a high applied electric field, which makes the polarization in the AFE layer approach the saturation state after only a few pulses. Consequently, the available current increment per pulse gradually decreases, leading to a loss of linearity in synaptic weight updates. To further evaluate complete long-term plasticity, both LTP and long-term depression (LTD) pulse schemes are applied. Ultimately, optimal linearity and symmetry are achieved using identical LTP pulses and tailored LTD pulses, yielding nonlinearity coefficient values of *α*_LTP_ = 0.35 and *α*_LTD_ =  − 2.09 (Fig. S11). Figure 4g shows the repeatedly measured LTP and LTD over 20 cycles using the determined pulse scheme; highly linear 7-bit synaptic responses with low cycle-to-cycle variation were obtained, suggesting the stability of the device for synaptic function emulation. Retention of multiple conductance levels is further confirmed in Fig. 4h. Ten distinct conductance states remain well-separated with no significant degradation over 10^3^ s, underscoring the robust nonvolatile performance of the device. In addition, a good endurance of over 10^4^ cycles is achieved, rendering the potential of the device for demonstrating reliable weight update (Fig. 4i). The D2D variation in nonvolatile behavior is also investigated in Figs. S12 and S13. A stable transfer characteristic with a large memory window is obtained across devices, confirming robust retention of the nonvolatile state. Furthermore, LTP measurements demonstrate a repeatable weight update, yielding an overall low D2D variation of 9% across all programmed states.

### Fading Memory in FG AFeFET for Physical Reservoir Demonstration

**Fig. 5:**
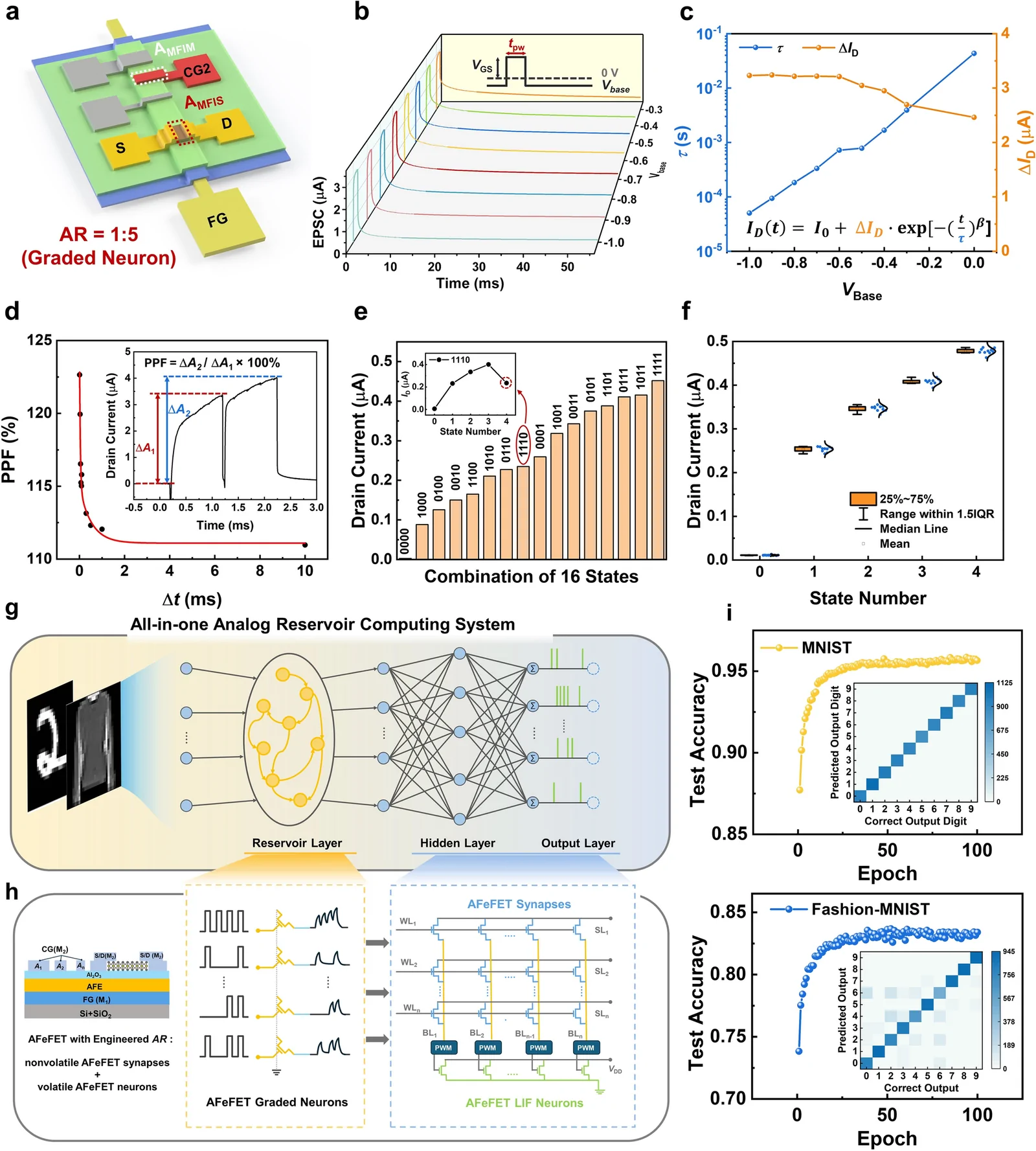
Fading memory characteristic of coplanar FG AFeFET with a medium AR and FG AFeFET-based all-in-one analog reservoir computing system. **a** Schematic demonstration that shows the activation of CG2 under the medium AR configuration. **b** Excitatory post-synaptic current as a function of time, the variation of base voltage *V*_base_ shows effective modulation on the current decay process. **c** Characteristic time constant *τ* and drain current Δ*I*_D_ extracted from the corresponding current decay process under varying *V*_base_ conditions. The negative shift of *V*_base_ results in a smaller *τ* and shows an acceleration effect on the current decay process, enabling multiple timescales to exist in a single device. **d** The inset shows paired-pulse facilitation (PPF) measurement of the FG AFeFET with *V*_set_ = 5.0 V, pulse width 1 ms, and *V*_base_ fixed at − 0.6 V. The corresponding PPF index is extracted from the measurement results. **e** Experimental readout current of the FG AFeFET in response to 16 pulse sequences that represent the 4-bit input signals (*V*_base_ is fixed at − 0.6 V). **f** Evolution of readout current under the stimulation of "1111", ten consecutive cycles are applied, with low cycle-to-cycle variation obtained. **g** Schematic of the all-in-one FG AFeFET-based analog reservoir computing (ARC) system. **h** Proposed hardware implementation for the demonstrated ARC system. **i** Test accuracies and corresponding confusion matrices for MNIST dataset and Fashion-MNIST dataset recognition tasks

In the ARC system, the fading memory and nonlinear dynamics serve as pivotal properties for the system to perform temporal summation and compress abundant sequential data, enabling efficient signal fusion. To emulate such behaviors, the AR of the device was further engineered to a medium value of 1:5 by adjusting the CG/FG overlapping area *A*_MFIM_ (Fig. 5a). Within this regime, the device exhibits a transient response that lies between purely volatile and fully nonvolatile operation, resulting in a semi-retentive characteristic. To evaluate this transition, a single programming pulse (1 ms, 5 V) was applied to the CG, followed by a read operation at *V*_GS_ = 0 V. As shown in Fig. S14, the device exhibits current integration during the applied pulse and a gradual current decay that stabilizes at a non-zero level after voltage removal, demonstrating a hybrid response shaped by dipole depolarization and weak memory effects.

This graded current integration and decay aligns with key features of physical reservoirs, namely nonlinearity and fading memory. Nonlinear current integration enables the nonlinear transformation of the input signals into high-dimensional space, and the gradual decay facilitates the internal connections between the input signals in the recent past, which assist in clarifying the underlying correlation between temporal inputs [44, 45]. Moreover, its weak memory effect provides additional flexibility in physical reservoir design. As shown in Fig. 5b, an additional modulation parameter “base voltage (*V*_base_)” is applied to the control gate to finely tune the decay characteristic of the residual *I*_D_ during the read process, thereby enabling controlled adjustment of the device dynamics. With the application of this small negative base voltage, the polarized dipoles originally retained during the read process are gradually forced to switch back, resulting in an accelerated recovery of the drain current. By modulating the amplitude of the *V*_base_, the drain current *I*_D_ relaxation speed varies distinctly, and a more negative *V*_base_ causes faster relaxation. To quantify this modulation effect, *I*_D_ was fitted using a stretched exponential model as expressed in Eq. 2 to extract the responsive current Δ*I*_D_ and characteristic time constant *τ* corresponding to varying *V*_base_ conditions.

2 $$I_{{\mathrm{D}}} \left( t \right) = I_{0} + \Delta I_{{\mathrm{D}}} \cdot \exp \left[ { - \left( { \frac{t}{\tau } } \right)^{\beta } } \right]$$

where *I*_0_ is the rest state of the current relaxation and *β* is the stretch component. From the fitting results in Fig. 5c, the characteristic time constant *τ* decreases by three orders of magnitude as *V*_base_ decreases from 0 to − 1 V, while Δ*I*_D_ exhibits only a small variation. This indicates that the temporal dynamics of the device can be freely controlled to meet the needs of different systems containing temporal information across multiple timescales. This characteristic greatly improves the design efficiency of the physical reservoir, as most physical reservoirs implemented by electronic devices usually possess a fixed timescale that is determined once the fabrication process is completed, which makes them only applicable for specific tasks [46, 47]. Furthermore, by exploiting these tunable temporal dynamics, the system can generate a rich set of reservoir states without compromising processing speed or increasing hardware complexity [48, 49]. This represents a significant advantage over conventional approaches that depend on large parallel reservoir arrays or deep cascaded architectures to enhance state diversity [50, 51], as further discussed and benchmarked in Table S3. In addition, the fitting parameter *β* shows no obvious dependence on the *V*_base_, which suggests the dominating influence of *V*_base_ on the time constant *τ* (Fig. S15). The fading memory property is further validated via paired-pulse facilitation (PPF) measurements. The second pulse induces a stronger *I*_D_ response compared to the first one, and this enhancement gradually diminishes with increasing intervals between the two pulses (inset of Fig. 5d). The extracted PPF indices fit a double exponential decay model, and the obtained time constant (*τ*_1_ = 34.6 µs, *τ*_2_ = 533 µs at *V*_base_ =  − 0.6 V) is consistent with the result extracted from EPSC measurement (Fig. 5c). To further confirm its ability to function appropriately as an effective physical reservoir, reservoir states separability was evaluated. Figure S16 demonstrates the dynamic evolution of the device response to the 4-bit sequential input sequences. Distinct reservoir states corresponding to 16 different combinations of input sequences are obtained by sampling the response current at the end of each input combination (Fig. 5e). The well-separated reservoir states demonstrate the high capability of the FG AFeFET for input signal encoding. Additionally, the cycle-to-cycle uniformity of the device in response to a specific input sequence is verified. As shown in Fig. 5f, the input sequence “1111” is repeatedly sent into the device for 10 consecutive cycles with identical pulsing parameters (*V*_GS_ = 5 V, *V*_base_ =  − 0.6 V, pulse width = 500 µs, interval = 500 µs). To statistically evaluate the state separability of the coplanar AFeFET-based reservoir, cycling tests were performed for all 16 input sequences (Fig. S17), thereby accounting for both cycle-to-cycle variation and unavoidable measurement noise. The resulting reservoir states were plotted as cumulative probability distributions (Fig. S18), revealing clearly distinguishable states with no apparent overlap. As summarized in Table S4, the small cycle-to-cycle variation and strong state separability demonstrate reliable input discrimination and a reduced processing error, marking a substantial improvement over conventional physical RC systems.

### All-in-One FG AFeFET-Based Analog Reservoir Computing System

Through effective AR engineering utilizing a coplanar structure and multigate design, both volatile, fading memory, and nonvolatile behaviors can be realized in a single FG AFeFET by selectively activating different control gates that define distinct AR configurations. Leveraging these diversifying functionalities, an all-in-one ARC system is constructed based on coplanar FG AFeFETs. As shown in Fig. 5g, all components inside the system can be realized with a multigate coplanar FG AFeFET structure, the volatile leaky–integrate–fire characteristic obtained under a large AR configuration is used to implement LIF neurons, the fading memory characteristic obtained under a medium AR configuration is adopted for physical reservoir implementation, and the nonvolatile memory characteristic obtained under a small AR configuration is utilized for synaptic weight update in the readout layer.

Pattern recognition tasks are performed using two different datasets to thoroughly assess the performance of the system, including the MNIST dataset containing hand-written digits and the Fashion-MNIST dataset consisting of different clothing patterns. In both datasets, each image containing 28 × 28 pixels is preprocessed by segmenting rows into seven 4-pixel blocks. Each block is binarized into a 4-time-step pulse sequence, which is then fed into the FG AFeFET-based reservoir layer. The device response at the last time step is collected as the reservoir states and transmitted into the next layer. With the application of this encoding method, each input image is scaled down to a size of 7 × 28 pixels while maintaining the spatial information. Next, the encoded information is input into a 196 × 128 × 10 fully connected readout network for processing, and the ten output neurons in the output layer generate distinct outputs based on the rate coding method. To closely emulate the practical system performance, experimentally extracted parameters from the device, such as reservoir responses to 4-bit inputs, multilevel synaptic weights, and the time constant *τ* of the LIF neuron, were incorporated into the simulation. In addition, a schematic demonstration of corresponding hardware implementation using coplanar FG AFeFET for the designed ARC system is illustrated in Fig. 5h. Within the system, the preprocessed input images first go through the FG AFeFET-based physical reservoir layer (medium AR configuration) for transformation. The transformed signals are then fed into a readout network composed of FG AFeFET-based synaptic devices (small AR configuration), where the bit-line current of the synaptic array is subsequently transmitted to FG AFeFET-based LIF neurons (large AR configuration) for spike generation. The system achieves recognition accuracies of 95.6% on the MNIST dataset and 83.4% on the Fashion-MNIST dataset, with confusion matrices presented in Fig. 5i. The reduced accuracy for the Fashion-MNIST dataset is attributed to its greater intrinsic complexity in both interclass and intraclass image variations compared to the MNIST dataset [52]. While this demonstration of the ARC system serves as a proof-of-concept, the modular and multifunctional nature of the device offers high design flexibility, enabling seamless integration of diverse neural network functionalities in a unified platform. The combination of high state separability, low energy consumption, and strong thermal stability (Fig. S19) underscores the suitability of this approach for the physical realization of large-scale ARC systems. A detailed discussion of the integration prospects and application potential of the coplanar FG AFeFET for scalable neuromorphic computing hardware is provided in Note S4 and Fig. S20. Moreover, the device’s key performance metrics against previously reported ferroelectric transistors are benchmarked in Table S5. Besides the demonstrated pattern recognition tasks, the coplanar FG AFeFET-based ARC system can also be adopted for processing dynamic and complex tasks. In Fig. S21, a time series prediction task is also conducted to provide a validation of its capability for temporal information processing.

## Conclusions

In this work, we have demonstrated a coplanar floating-gate antiferroelectric field-effect transistor that achieves tunable memory behavior through structural design and area ratio engineering. By employing independent control gates, the device consolidates three distinct neural functionalities within a single unit, including volatile leaky–integrate–fire neuron dynamics, physical reservoir responses, and nonvolatile synaptic characteristics. The LIF mode accommodates both excitatory and inhibitory stimuli while exhibiting intrinsic self-resetting behavior, thereby eliminating the need for external reset circuitry. The reservoir mode enables distinct reservoir states and multi-timescale dynamics via *V*_base_ modulation, while the synaptic mode provides symmetric, linear conductance updates across 128 states (7-bit resolution). Beyond functional versatility, the coplanar architecture enhances the vertical scalability of conventional FG configurations, delivering both a large memory window and a high on/off ratio. The mechanisms underpinning these tunable functionalities are systematically elucidated through load line analysis and energy band diagrams. Harnessing these multifunctional capabilities, a complete ARC system is successfully realized within a unified coplanar FG AFeFET framework, achieving high recognition accuracies on the MNIST and Fashion-MNIST datasets. These results establish the coplanar FG AFeFET as a promising building block for compact, scalable, and energy-efficient neuromorphic computing systems.

## Supplementary Information

Below is the link to the electronic supplementary material.

Supplementary file1 (DOCX 11959 kb)
