# Supplementary material for: Coplanar Floating-Gate Antiferroelectric Transistor with Multifunctionality for All-in-One Analog Reservoir Computing
Source: Nanomicro Lett. 2026 Jan 8;18:202. doi: 10.1007/s40820-025-02049-9 (PMC12783432; doi:10.1007/s40820-025-02049-9)
Supplement: Supplementary file 1 — Supplementary file1 (DOCX 11959 kb) [file 40820_2025_2049_MOESM1_ESM.docx]

Supporting Information for

Coplanar Floating-gate Antiferroelectric Transistor with Multifunctionality for All-in-one Analog Reservoir Computing

Yufei Shi^1^, Zijie Zheng^1^, Jiali Huo^1^, Yu-Chieh Chien^1^, Sifan Li^1^, Haofei Zheng^1^, Xiao Gong^1^, and Kah-Wee Ang^1^*

^1^ Department of Electrical and Computer Engineering, National University of Singapore, 4 Engineering Drive 3, 117583, Singapore

*Corresponding author. E-mail: [eleakw@nus.edu.sg](mailto:eleakw@nus.edu.sg) (Kah-Wee Ang)

**Note S1** **Equivalent Capacitance Model of Coplanar FG AFeFET**


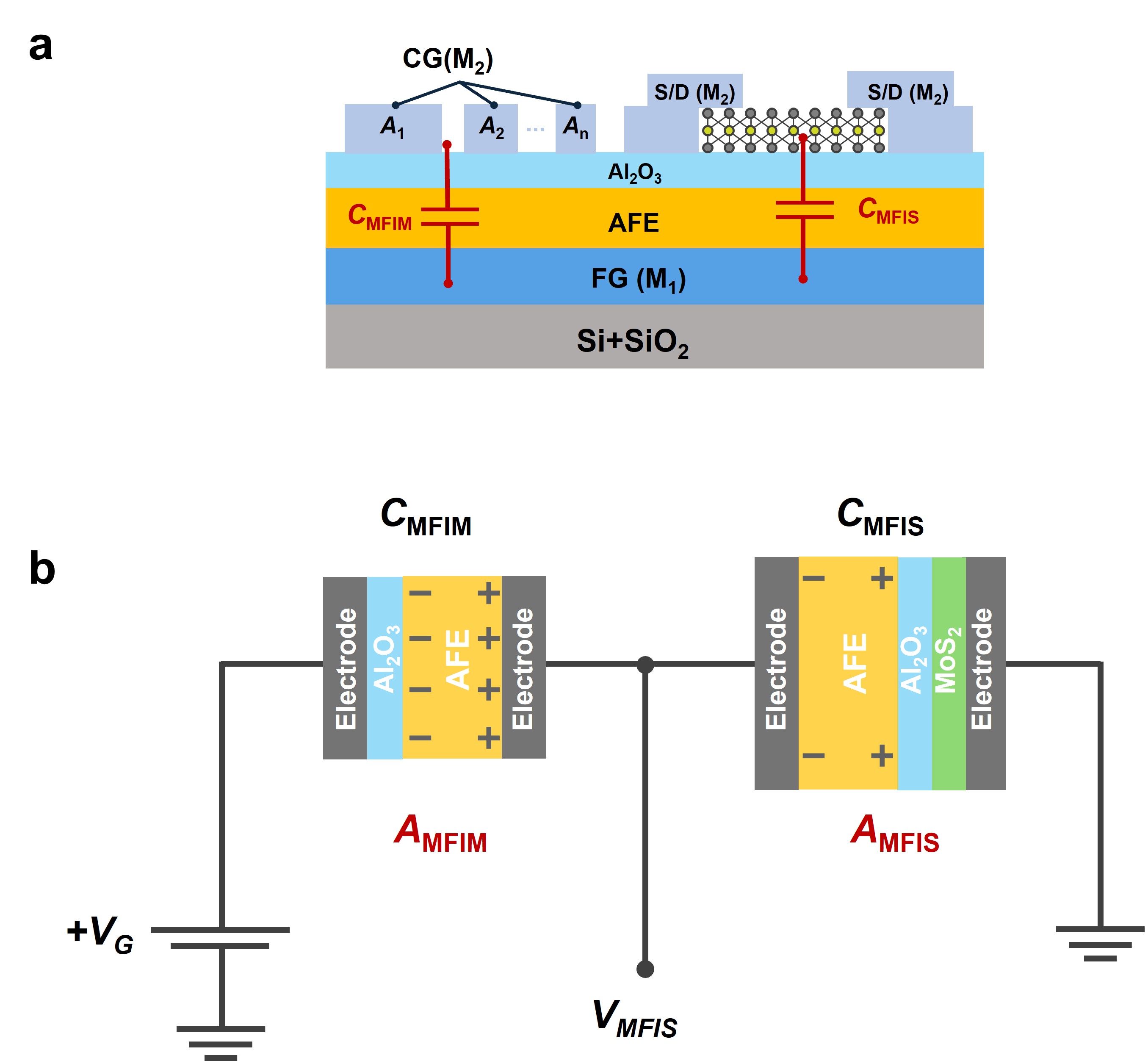


**Fig. S1 a** Schematic demonstration of existing capacitance within the coplanar FG AFeFET. **b** The proposed equivalent capacitance model of coplanar FG AFeFET gate stack. Inside the model, the components between the control gate and floating gate are modeled using *C*_MFIM,_ and the overlapping area between the two electrodes is *A*_MFIM_. The components between the floating gate and source & drain terminals are modeled using *C*_MFIS_, and the overlapping area is *A*_MFIS._

To get a clear view of the gate voltage division across the coplanar FG AFeFET gate stack during operation, an equivalent capacitance model is proposed, as shown in **Figure S1b**. Inside the model, the structure between the control gate and floating gate (MFIM region) is modeled as *C*_MFIM_, comprising the series connection of antiferroelectric Hf_0.25_Zr_0.75_O_2_ layer capacitance *C*_HZO_ and Al_2_O_3_ insulating layer capacitance *C*_ins_. Similarly, the structure between the CG and source & drain terminal (MFIS region) is modeled as *C*_MFIS_, including a series connection of *C*_HZO_, *C*_ins,_ and capacitance of 2D semiconducting channel *C*_S._

$$C_{\mathrm{MFIM}}={(\frac{1}{C_{HZO}}+\frac{1}{C_{ins}})}^{-1}$$

$$C_{MFIS}={(\frac{1}{C_{HZO}}+\frac{1}{C_{ins}}+\frac{1}{C_{S}})}^{-1}$$

Additionally, the coplanar FG architecture allows effective AR engineering inside the device. Therefore, the capacitance model also needs to consider the area effect. By considering an area of *A*_MFIM_ and *A*_MFIS_ for the MFIM and MFIS region respectively, the *C*_MFIM_ and *C*_MFIS_ can be further expressed as:

$$\frac{1}{C_{MFIM}}=\frac{1}{\varepsilon_{0}\cdot A_{MFIM}}(\frac{d_{HZO}}{\varepsilon_{HZO}}+\frac{d_{ins}}{\varepsilon_{ins}})$$

$$\frac{1}{C_{MFIS}}=\frac{1}{\varepsilon_{0}\cdot A_{MFIS}}(\frac{d_{HZO}}{\varepsilon_{HZO}}+\frac{d_{ins}}{\varepsilon_{ins}}+\frac{d_{S}}{\varepsilon_{S}})$$

When a gate voltage *V*_GS_ is applied:

$V_{GS}=V_{MFIS}+V_{MFIM}$, $\frac{V_{MFIS}}{V_{MFIM}}= \frac{C_{MFIM}}{C_{MFIS}}$

Therefore, $V_{MFIS}= V_{GS}\cdot\frac{C_{MFIM}}{C_{MFIS}+C_{MFIM}}$, $V_{MFIM}= V_{GS}\cdot\frac{C_{MFIS}}{C_{MFIS}+C_{MFIM}}$

$$\frac{V_{MFIM}}{V_{MFIS}}= \frac{A_{MFIS}}{A_{MFIM}}\cdot\frac{\frac{d_{HZO}}{\varepsilon_{HZO}}+\frac{d_{ins}}{\varepsilon_{ins}}}{\frac{d_{HZO}}{\varepsilon_{HZO}}+\frac{d_{ins}}{\varepsilon_{ins}}+\frac{d_{S}}{\varepsilon_{S}}} \approx\frac{A_{MFIS}}{A_{MFIM}}$$

From the above derivation, it is evident that the voltage division across the MFIS and MFIM regions is mainly determined by the area ratio between these two regions (AR = *A*_MFIM_/*A*_MFIS_). More importantly, different from the complex voltage distribution relationship of conventional FG FE/AFeFET, the voltage division ratio directly follows an inverse relationship with the area ratio, indicating the effective modulation effect of AR engineering on device performance by using the designed coplanar structure. It should be noted that, in a practical case, the dielectric constant of the HZO layer at the MFIM and MFIS regions may vary according to the actual voltage drop on these two regions. Here $\varepsilon_{HZO\_MFIM}(V_{MFIM})$ and $\varepsilon_{HZO\_MFIS}(V_{MFIS})$ are both simplified to a fixed constant $\varepsilon_{HZO}$ to simplify the derivation process.


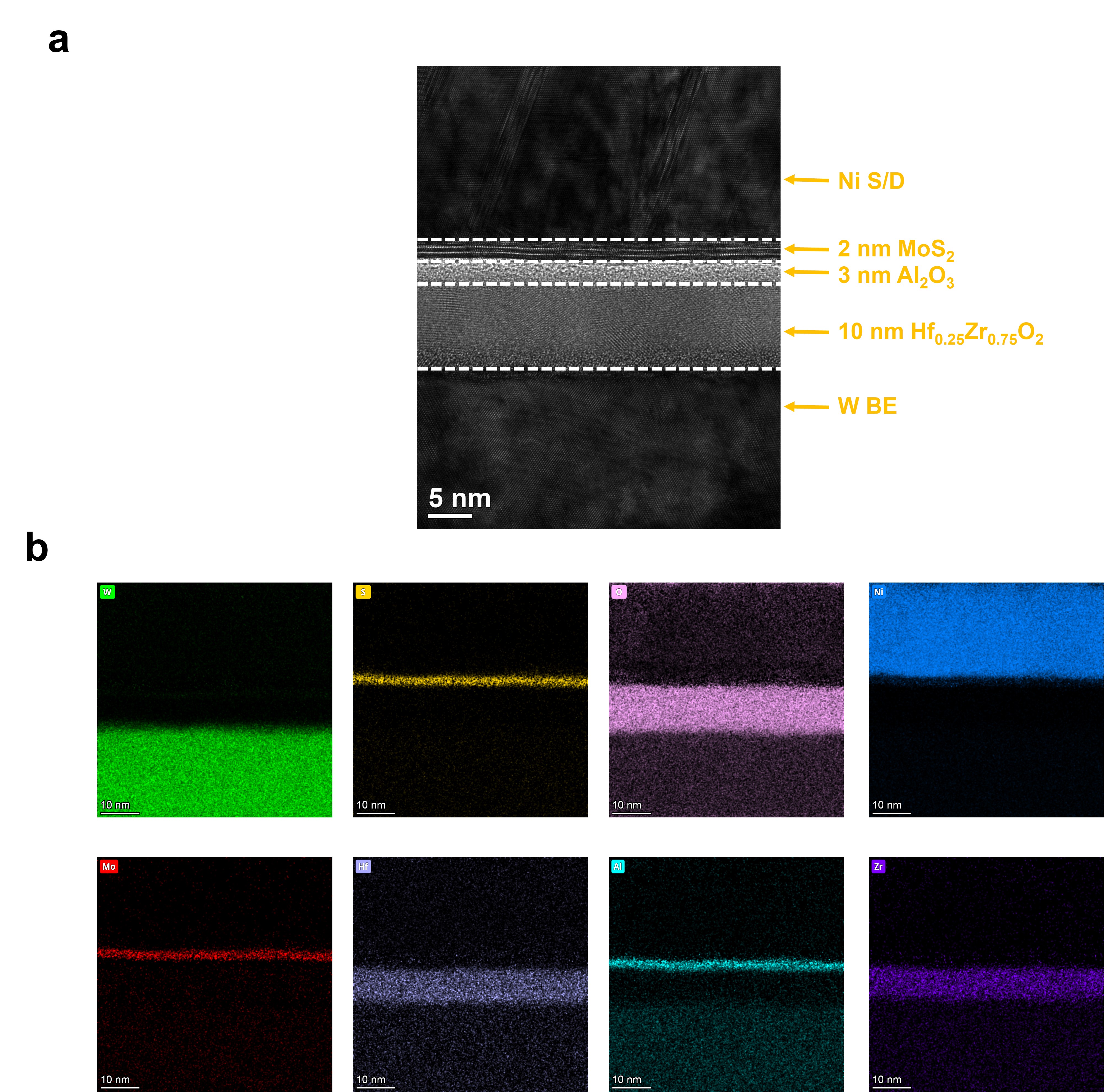


**Fig. S2** **a** High-resolution TEM cross-sectional image of the source/drain region. **b** EDS elemental mapping of the coplanar FG AFeFET, showing clear interfaces between different layers.


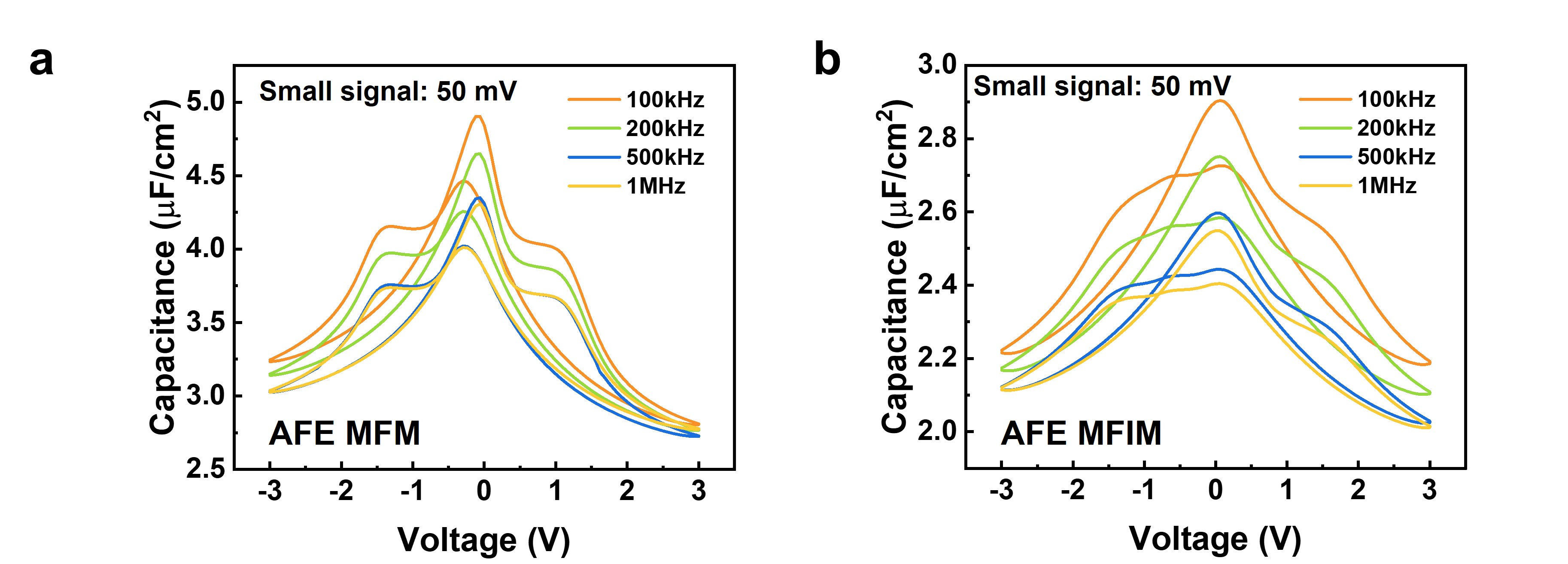


**Fig. S3 a** Capacitance-voltage (*C*-*V*) curves of the MFM capacitor under various small signal frequencies. A high dielectric constant of ~50 is extracted when measured at 100 kHz. **b** *C*-*V* curves of the MFIM capacitor under various small signal frequencies. Under a dual voltage sweep, the *C*-*V* curves of the MFIM capacitor no longer show a standard double-humped curve with four capacitance peaks possessed by the AFE MFM capacitor. Differently, its *C*-*V* curve shows a transitioning trend towards the butterfly loop of the FE MFM capacitor. The above results suggest that the insertion of the additional Al_2_O_3_ layer in the AFE MFM capacitor enhances the AFE- FE phase transition under the applied voltage.

It is worth noting that, with the insertion of an additional 3 nm Al_2_O_3_ layer, an obvious increase in the remanent polarization *P*_r_ can be observed, and the polarization-voltage curve transitions towards a ferroelectric-like behavior, showing a single hysteresis loop instead of the typical double hysteresis loop of an antiferroelectric. In addition, the displacement current obviously reduces because of the presence of the Al_2_O_3_ layer.


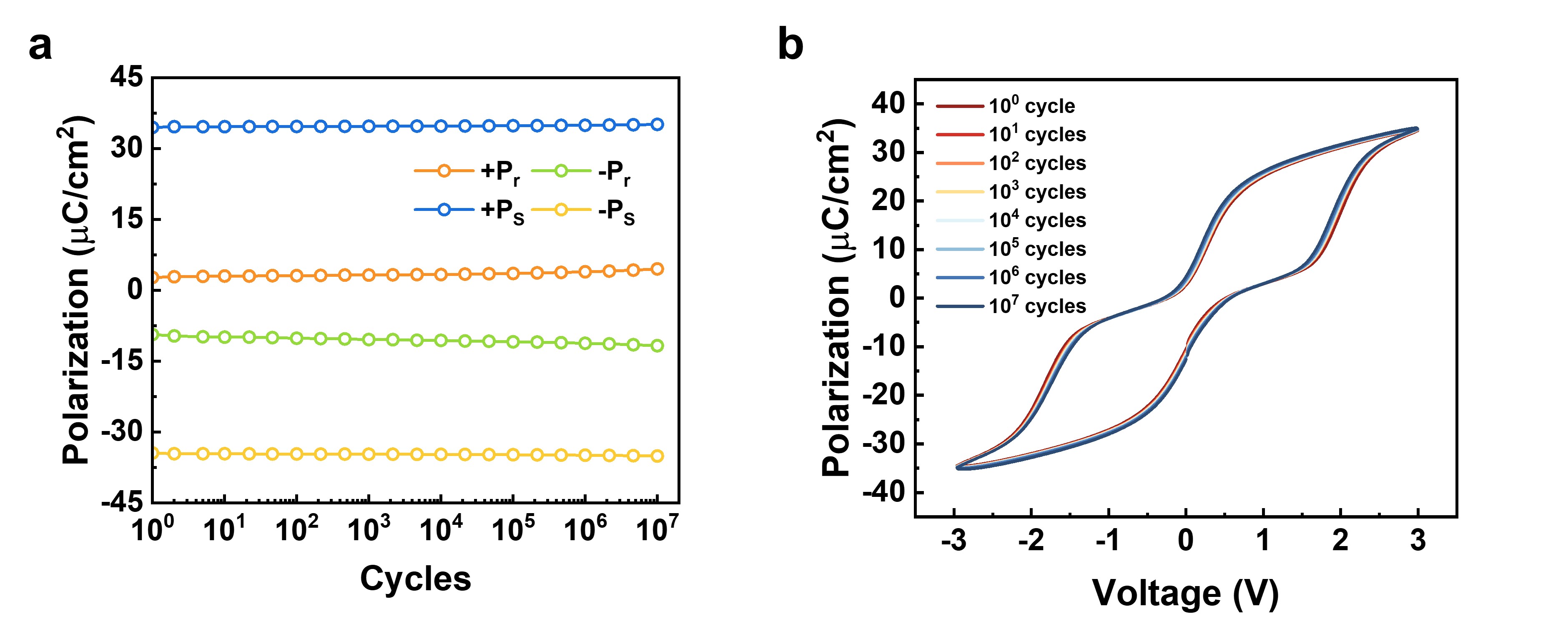


**Fig. S4** Endurance measurement of AFE Hf_0.25_Zr_0.75_O_2_ MFM capacitor. **a** The change of *P*_s_ and *P*_r_ during 10^7^ cycling pulses with a frequency of 1MHz. **b** Evolution of *P*-*V* hysteresis loop during cycling test.

The long-term reliability of the AFE phase in Hf_0.25_Zr_0.75_O_2_ film is a critical consideration for practical use, which is governed by several influencing factors. First, accurate control of the composition and thickness of the AFE HZO film is vital for achieving a reliable AFE phase [S1]. It is the basis for obtaining long-term AFE behavior, and the drift of composition or film thickness may cause the transition of the AFE phase into the paraelectric or ferroelectric phase. In addition, interface quality and selection of metal electrodes are also determining factors [S2]. The stress induced by top/bottom metal electrodes usually modifies the free-energy landscape of the AFE HZO film and therefore influences the AFE phase stability. And interfacial quality and defect density are also important, as the redistribution of the interfacial charges and oxygen vacancies inside the film can cause the degradation of the AFE phase. Besides these factors related to the intrinsic material, the measurement condition also plays a vital role in controlling AFE phase stability [S3]. High cycling field and asymmetric cycling pulse are detrimental to AFE phase stability, while a moderate or low bipolar pulse is preferred. Moreover, an even better endurance characteristic can be obtained if the bipolar stressing pulse is further replaced by the unipolar stressing pulse [S4]. In this work, the AFE Hf_0.25_Zr_0.75_O_2_ layer operates under a moderate applied electric field. In this way, the device is capable of maintaining long-term phase stability under repeated cycling and elevated-temperature operation.


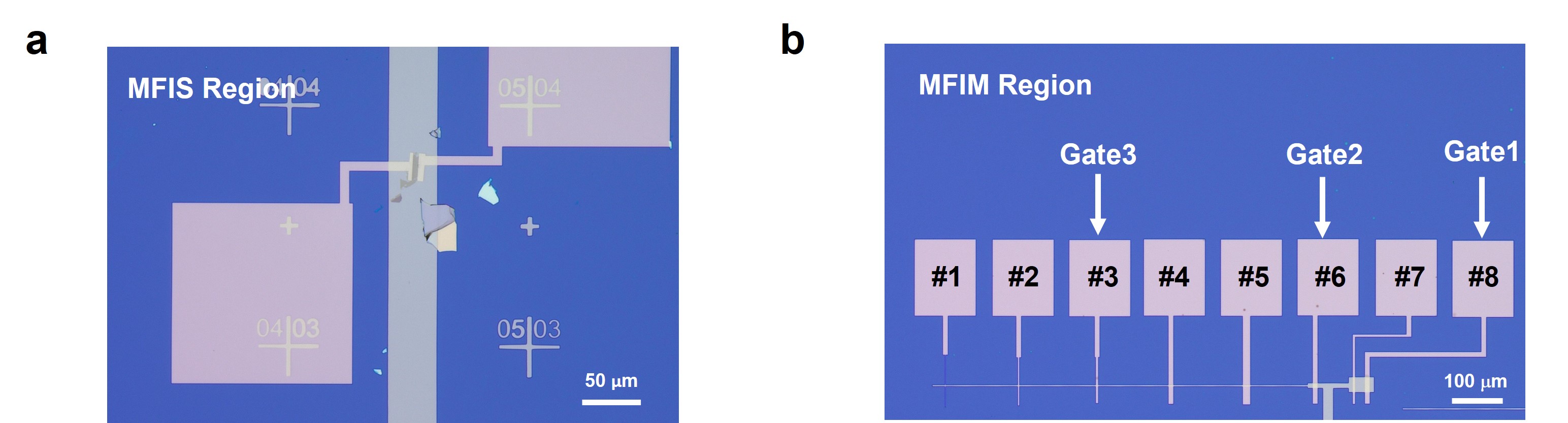


**Fig. S5** Optical microscope image of the coplanar FG AFeFET. **a** Image of channel region, the overlapping area between source/drain electrodes and floating gate determines the area of MFIS region *A*_MFIS_. **b** Image of MFIM region with multiple control gates featuring various *A*_MFIM_ designs. The actual dimensions corresponding to different parameters are listed in the following tables.

**Table S1** Actual dimensions of key parameters in the MFIS region

| **MFIS Parameter** | **Value** |
| --- | --- |
| Channel Length L | 4.4 µm |
| Channel Width W | 23.3 µm |
| Source Electrode Overlap A_1_ | 201.6 µm^2^ |
| Drain Electrode Overlap A_2_ | 206 µm^2^ |
| Area of MFIS: *A*_MFIS_ = W*L + A_1_+ A_2_ = 510.1 µm^2^ | |

**Table S2** Actual dimensions of key parameters in the MFIM region

| **MFIM Parameter** | **Value** | **Area Ratio** |
| --- | --- | --- |
| #1 Gate Area | 6 µm^2^ | 1:85 |
| #2 Gate Area | 9 µm | 1:56.6 |
| #3 Gate Area | 15 µm^2^ | 1: 34 |
| #4 Gate Area | 30 µm^2^ | 1:17 |
| #5 Gate Area | 45 µm^2^ | 1:11.3 |
| #6 Gate Area | 100 µm^2^ | 1:5 |
| #7 Gate Area | 150 µm^2^ | 1:3.4 |
| #8 Gate Area | 450 µm^2^ | 1:1.2 |

**Note S2 Basis of Load Line Analysis**

The load line analysis is employed in Fig. 3b and Fig. 4b to investigate the dynamic evolution of the device’s operating points under different applied voltages for two different area ratio conditions (AR = 1:1 and AR << 1), and the analytical results show high consistency with the measured *I*_D_-*V*_GS_ curves. Here, the basis related to this graphical analysis method is provided [S5].

During the analysis, the entire device is modeled as a serial connection of an MFIM capacitor and MFIS FET, representing the MFIM and MFIS regions, respectively. The target of the analysis is to use their charge-voltage (*Q*-*V*) curves to qualitatively determine the drain current of the device at different given *V*_GS_ and subsequently analyze its possible operating point. The physical basis of the analysis relies on Gauss’s law and can be summarized into the following equations:

$V_{MFIM}=V_{GS}-V_{MFIS}$ (S1)

${Q_{MFIM}(V}_{MFIM})={Q_{MFIS}(V}_{GS}-V_{MFIM})+Q_{Trap}$ (S2)

Where *Q*_MFIM_ and *Q*_MFIS_ represent the charge in the MFIM and MFIS region during device operation, and both of them are functions of the voltage drop in the corresponding regions. As various area ratios are designed in the device, the area of these two regions is also taken into consideration. In this way, the total charge in each part can be expressed as follows:

$Q_{MFIM}=A_{MFIM}\cdot q_{MFIM}$ (S3)

$Q_{MFIS}=A_{MFIS}\cdot q_{MFIS}$ (S4)

The *q*_MFIM_ and *q*_MFIS_ represent the total charge density of different regions. Combining the equation S1-S4 listed above and recall the definition of area ratio $AR=\frac{A_{MFIM}}{A_{MFIS}}$, the overall *Q*-*V* relationship of the device can be obtained:

${q_{MFIM}(V}_{MFIM})={q_{MFIS}(V}_{GS}-V_{MFIM})\cdot\frac{1}{AR}+\frac{Q_{Trap}}{A_{MFIM}}$ (S5)

Load line analysis serves as a graphical solution to the above equations using measured results. By plotting the *Q*-*V* curve of the MFIM capacitor and MFIS FET on the same plane and dynamically moving the corresponding lines based on the applied *V*_GS_, the generated intersection points will automatically satisfy the above equation and subsequently represent the operating point of the device.

The main principles for line movement in the load line graph are summarized below:

1. Effect of applied *V*_GS_: applying *V*_GS_ corresponds to the horizontal sliding of the ${Q_{MFIS}(V}_{GS}-V_{MFIM})$ in the load line graph.

2. Effect of AR engineering: when an unbalanced area ratio (AR ≠ 1) is designed, the area of the MFIM region *A*_MFIM_ will change accordingly to a larger value (when AR > 1) or smaller value (when AR < 1) than *A*_MFIS_. Based on the equation S5, its *Q* value under a certain voltage will increase and decrease by a factor of AR, respectively.


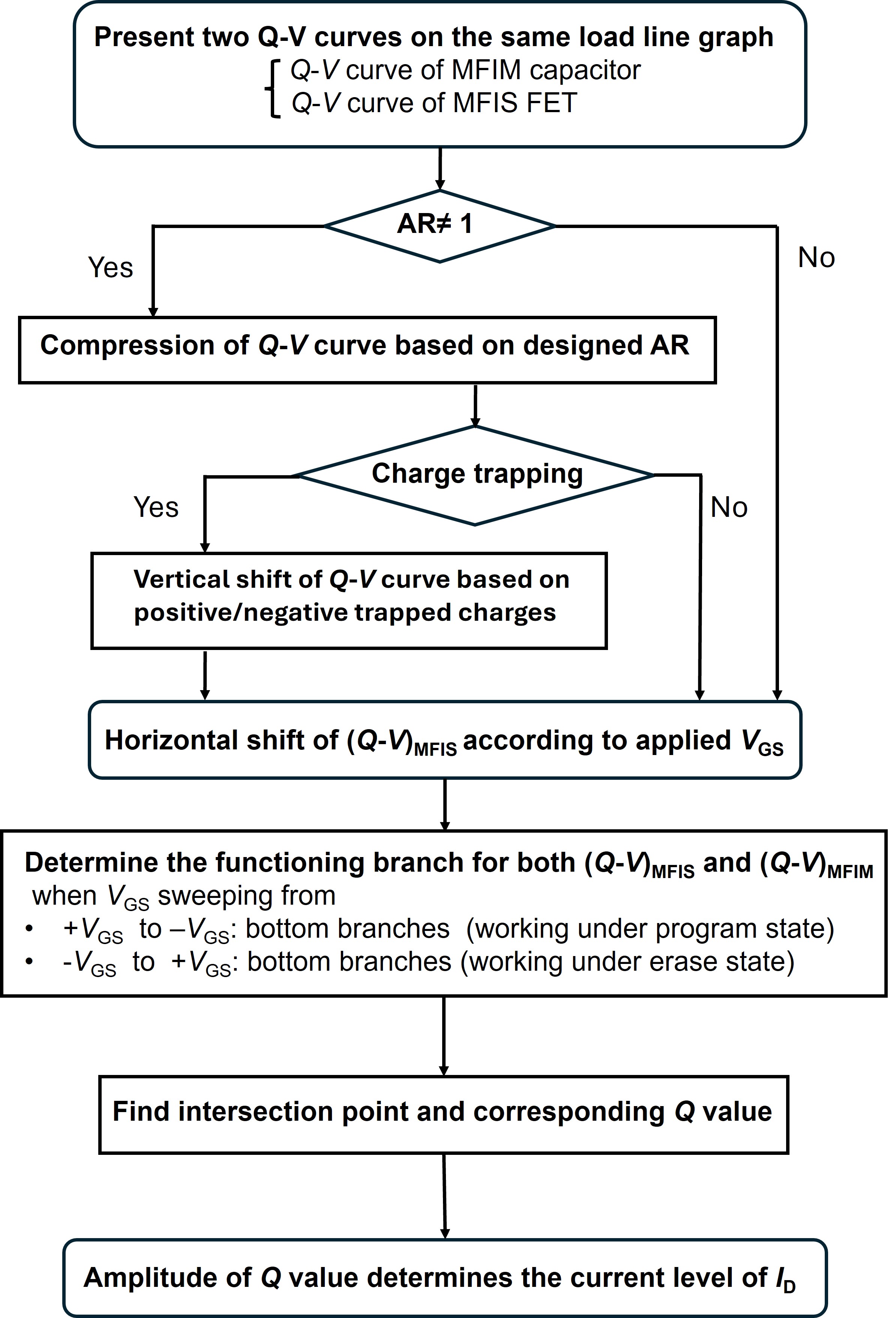


**Fig. S6** Flow chart demonstrated the detailed procedures of load line analysis

3. Effect of trapped charges: when charge trapping and detrapping presents, the influence of generated trapped charges should also be included in the analysis. Based on the equation S2,$Q_{MFIS}$ and ${Q_{MFIS}(V}_{GS}-V_{MFIM})$ should be plotted within the same plane. And the effect of *Q*_Trap_ is equivalent to the vertical sliding of the original line ${Q_{MFIS}(V}_{GS}-V_{MFIM})$. e.g. In our analysis, the *Q*_Trap_ is considered in Fig. 4b under an AR << 1 condition, and the positive charge trapping from the CG into FG (equivalent to electron detrapping from the FG) is included as the upshift of ${Q_{MFIS}(V}_{GS}-V_{MFIM})$ line, as indicated by the pink x-axis in the upper panel of Fig. 4b. A flow chart that shows the detailed procedures of the load line analysis is demonstrated in **Fig. S6** to provide an overview of this method.


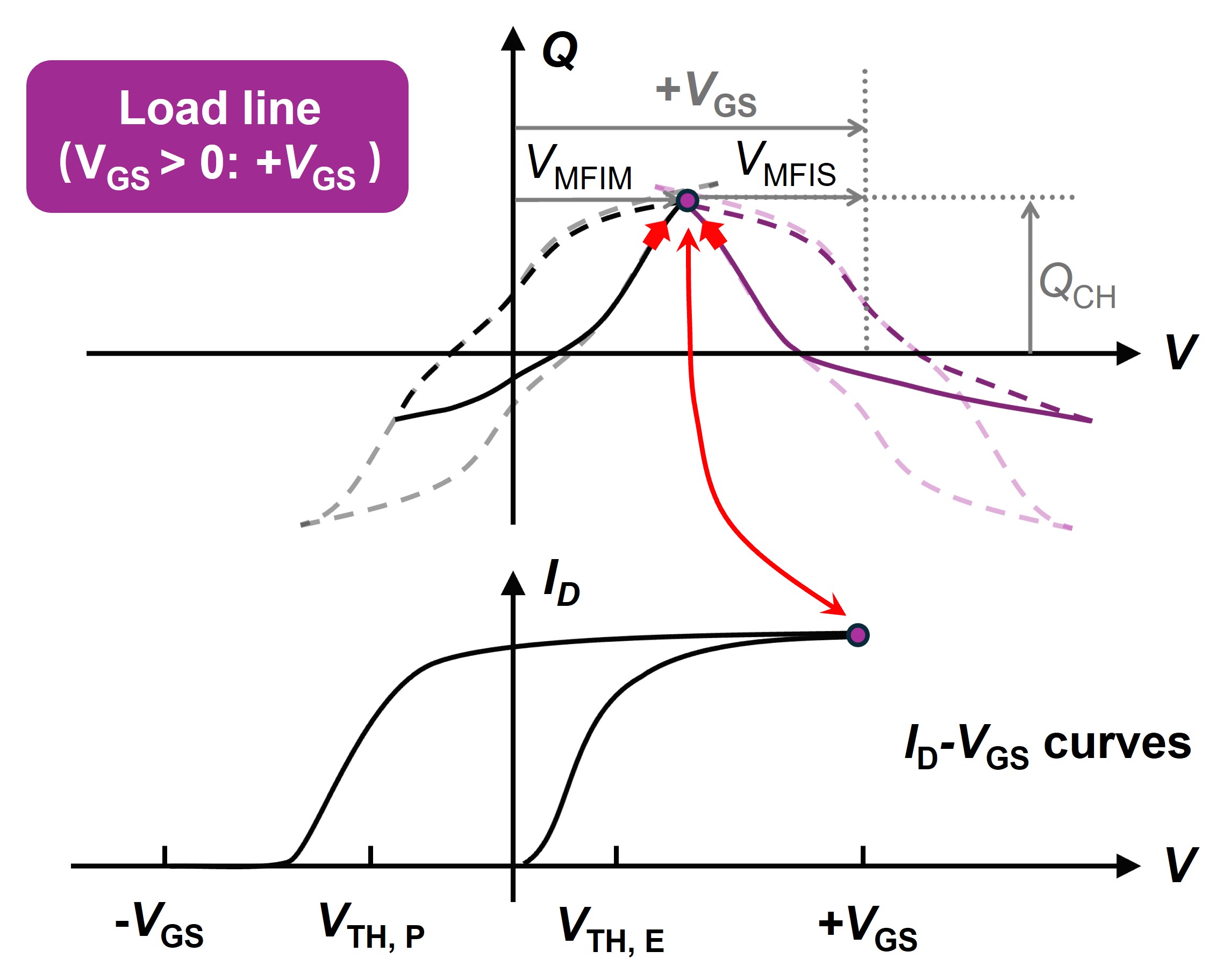


**Fig. S7** Load line analysis of coplanar FG AFeFET with AR = 1:1 under a positive applied gate voltage +*V*_GS_.

Here, we use **Fig. S7** as an example to explain the detailed process to find the operating point of the device under a positive applied gate voltage +*V*_GS_. As shown in Fig. S6, when the applied voltage increases from the initial *V*_GS_ = 0 V to +*V*_GS_, the *Q*-*V* line for MFIS FET is first horizontally shifted to a position where its center is at *V* = +*V*_GS_. As the initial state of *V*_GS_ is 0 V before applying +*V*_GS_, during this *V*_GS_ increase process, the functioning branch of the *Q*-*V* curves in both the MFIM capacitor and MFIS FET is the bottom branch, labelled using solid lines, while the standby branch is labelled using dashed lines. In this context, these two functioning lines will generate a single intersection point, which is the operating point we expect under applied +*V*_GS_. From the obtained operating point (labelled as a purple dot), a high *Q* value can be observed. It indicates the high drain current existed under +*V*_GS_, labelled as a purple dot in the *I*_D_-*V*_GS_ curve. By repeating similar analysis under different *V*_GS_ conditions, different operating points can be captured and a complete *I*_D_-*V*_GS_ can be generated theoretically. Detailed analysis information corresponding to the device operating at different *V*_GS_ is provided in **Fig. S9**.

**Note S3 Back-switching Characteristic in Intrinsic AFE Film**


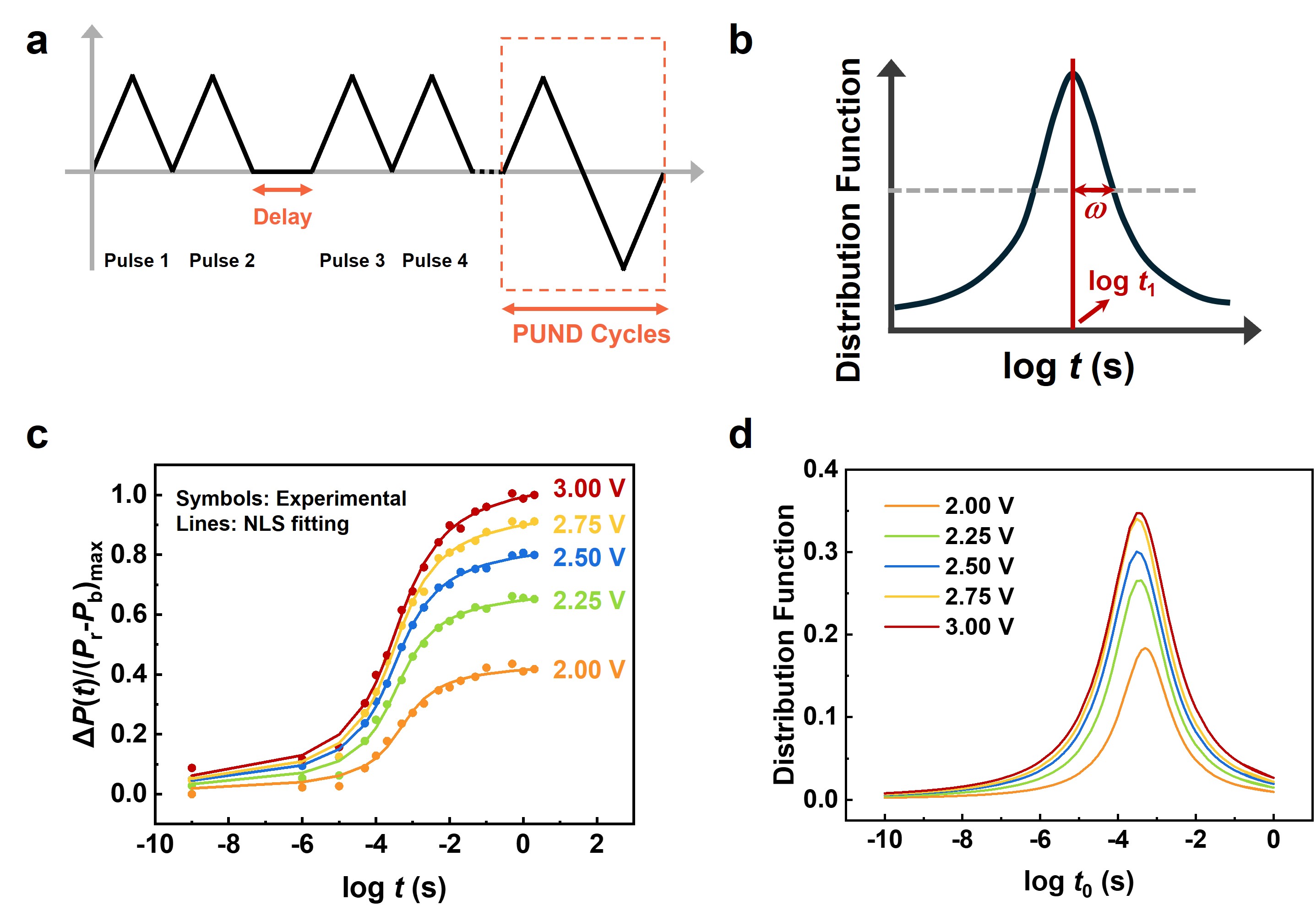


**Fig. S8** Back-switching kinetics of intrinsic AFE HZO film. **a** Schematic of the designed pulse sequence for back-switching kinetics measurement. **b** Schematic of Lorentzian distribution and related key parameters. **c** Time-dependent back-switched polarization (Δ*P*(*t*) = *P*_r_ − *P*_b_) under various external voltages. Scatters represent the experimental data, and the solid lines represent the results obtained from NLS fitting. **d** Lorentzian distributions corresponding to the fitting results in Fig. S8c.

To provide a more comprehensive characterization of the intrinsic physical properties of the AFE Hf_0.25_Zr_0.75_O_2_ layer on device performance, here we utilize the nucleation-limited-switching (NLS) model to investigate the polarization back-switching kinetics in the HZO layer, as it is pivotal in contributing to the fading memory property that is crucial for realizing the physical implementation. Based on the NLS model, the polycrystalline AFE HZO film is considered to be composed of multiple domains that can be switched independently; each of them possesses a distinctive domain back-switching time [S6, S7]. Therefore, the characteristic back-switching time *t*_0_ of the polycrystalline HZO film is no longer a single value but follows a Lorentzian distribution F(log*t*_0_), and the time-dependent polarization back-switching behavior inside the film is described by the NLS model as expressed by **Eq. S6**:

$$\frac{\Delta P\left( t \right)}{2P_{s}}=\int_{\infty}^{-\infty} \left[ 1-exp-\left( \frac{t}{t_{0}} \right)^{2} \right]F\left( logt_{0} \right)d\left( logt_{0} \right) (S6-1)$$

$$F(logt_{0})=\frac{A}{\pi}[\frac{\omega}{{(logt_{0}-logt_{1})}^{2}+\omega^{2}}] (S6-2)$$

where *A* is a normalization constant, *w* is the half-width at half-maximum and log*t*_1_ is the central value of the distribution.

**Figure S8a** shows the designed pulse sequence for back-switching kinetics measurement. During the measurement, the first pulse is applied to polarize all the dipoles in one direction, and the second pulse is added to record the change of non-switching polarization. Next, a delay period ranging from 1 μs to 2 s is inserted before the application of pulse 3 for spontaneous depolarization. Subsequently, the third pulse is applied to record the portion of the back-switching dipole, and pulse 4 is applied at the end to confirm no additional back-switched dipoles when the delay period is removed. For the fitting of the back-switching kinetics, Δ*P*(t) is calculated as (*P*_r_ - *P*_b_), and the parameter in Equation S6 is substituted by (*P*_r_ - *P*_b_)_max_, in which *P*_b_ is the smallest *P*_r_ value the material can achieve under a sufficiently long interval. As the delay period increases from 1 μs to 2 s, the recorded Δ*P*(t) increases gradually, indicating an increase in the number of back-switched dipoles. **Fig. S8c** shows the time-dependent back-switched dipoles of the 10 nm AFE HZO film under different applied voltages, the measured data are well-fitted by the NLS model, and the corresponding Lorentzian distribution F(log*t*_0_) is shown in **Fig. S8d**. In the fitted distribution functions, the value of log*t*_1_ only shows slight variation between -4 to -3, which represents a characteristic back-switching time of around hundreds of microseconds to 1 ms. This characteristic back-switching time obtained from the AFE HZO layer shows high consistency with the decay time constant obtained from the coplanar AFeFET. This result exhibits the decisive role of the intrinsic volatility of the AFE HZO layer on the volatile behavior of the device.


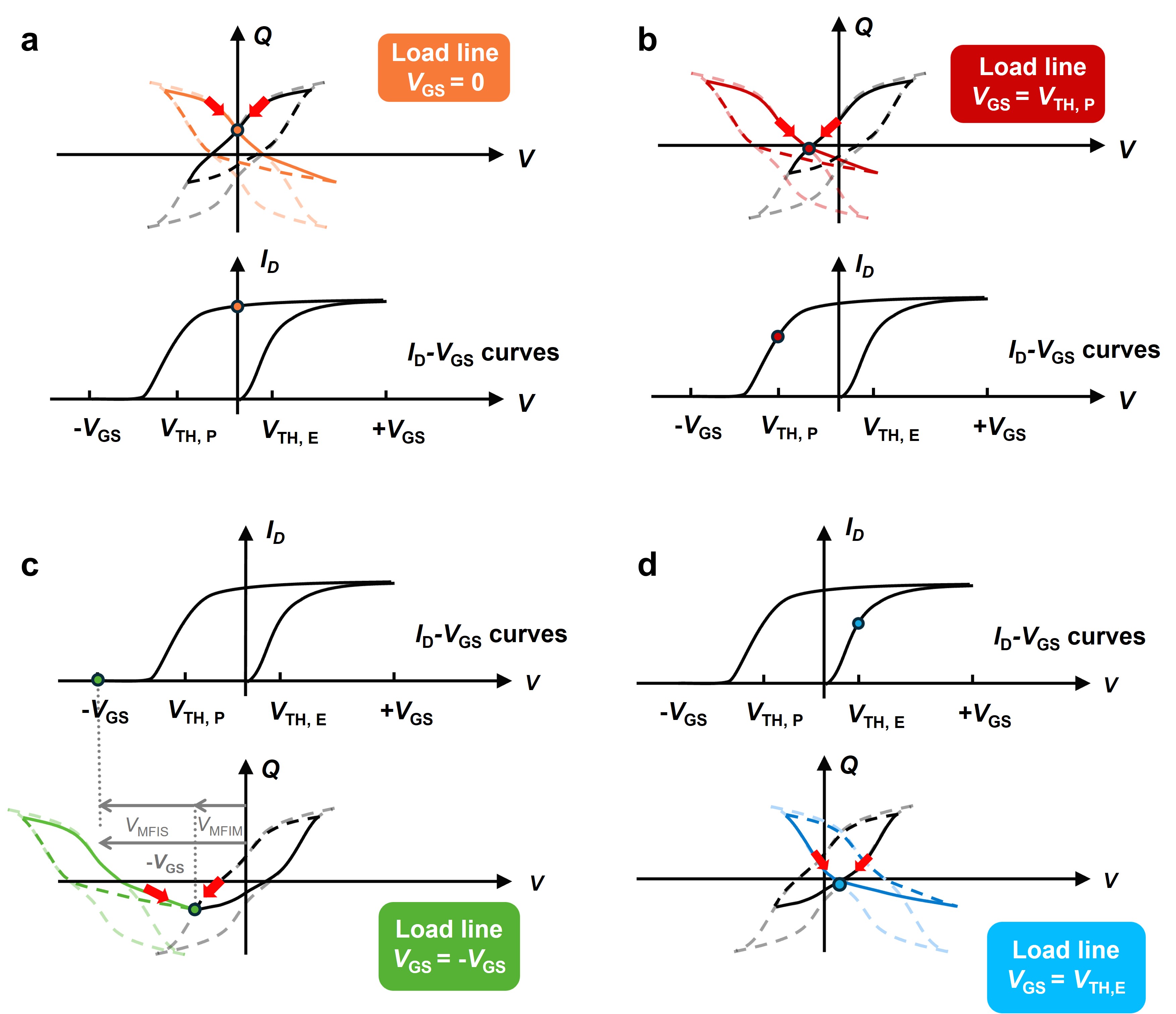


**Fig. S9** Detailed load line graphs by splitting the load line analysis of coplanar FG AFeFET with AR = 1 into different subsections, each of them corresponding to device operating at different *V*_GS._ **a** When *V*_GS_ = 0 V. **b** when *V*_GS_ = *V*_TH,P._ **c** when *V*_GS_ = −*V*_GS_. **d** when *V*_GS_ = *V*_TH,E_


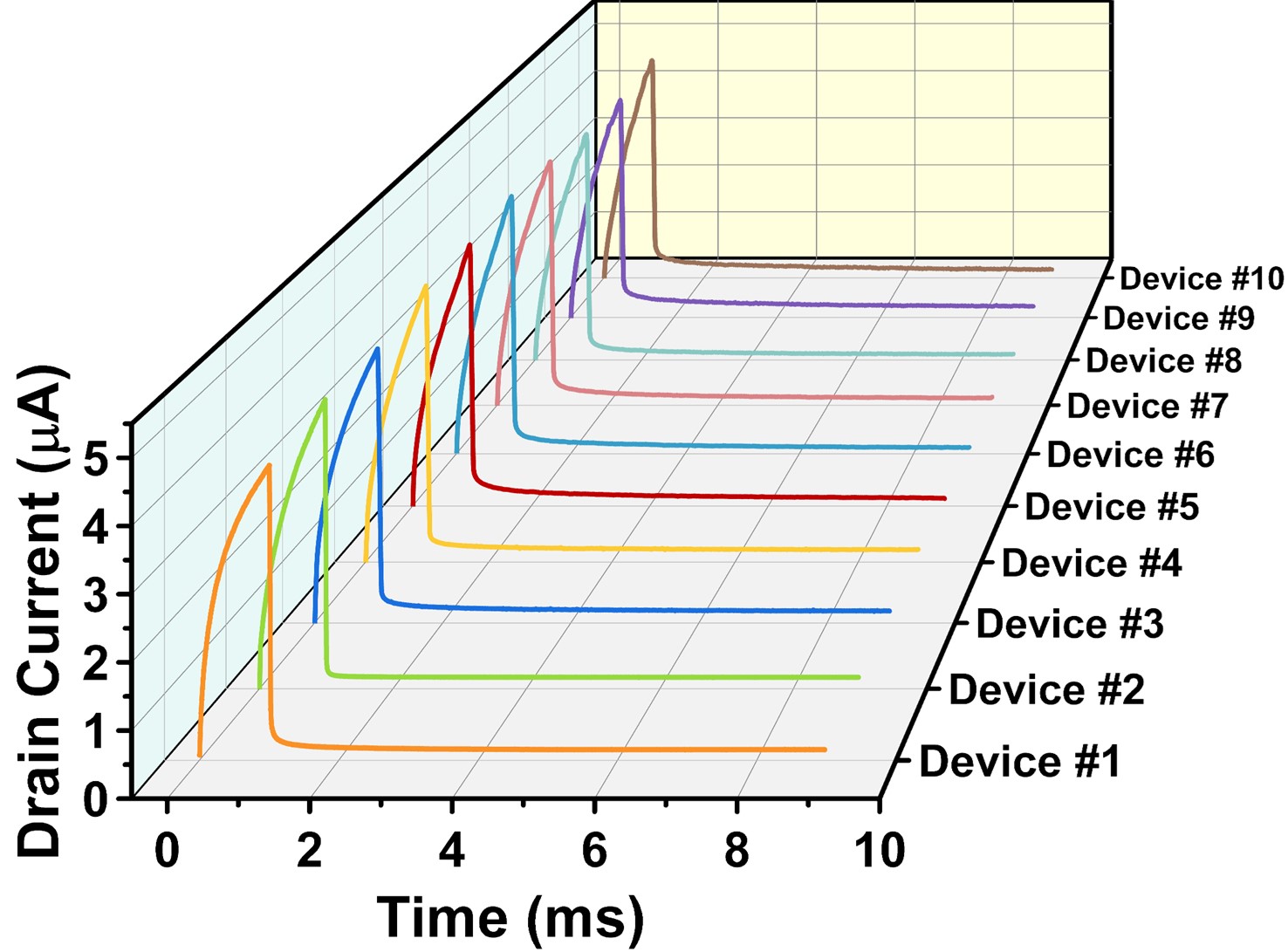


**Fig. S10** Device-to-device variation on volatile behaviors of the coplanar AFeFET across 10 devices


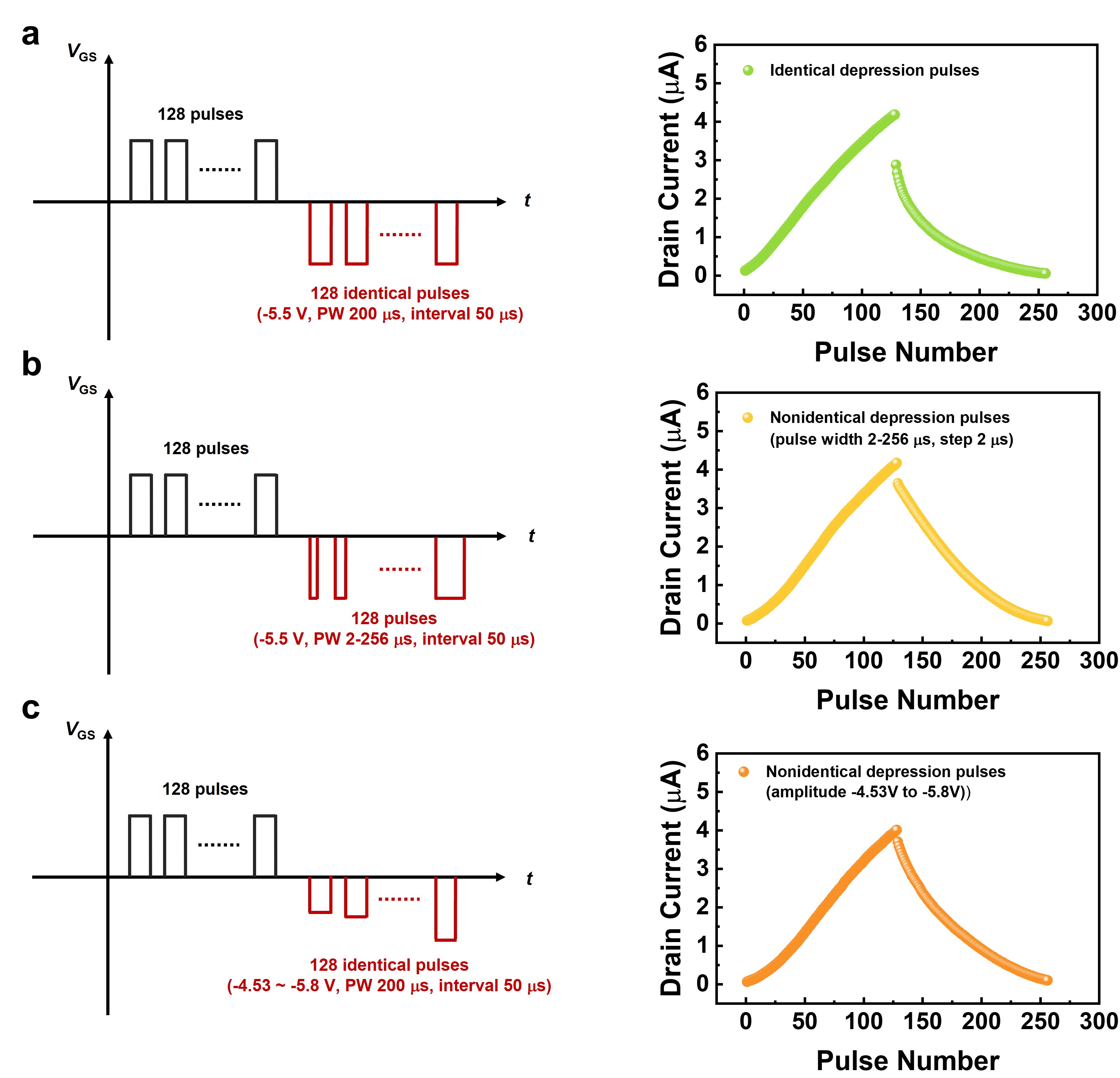


**Fig. S11** Long-term potentiation and long-term depression characteristics under different applied pulse schemes. All potentiation processes use the same identical pulse sequences (*V*_GS_ = 5.5 V, *t*_PW_ = 200 μs, interval = 50 μs), while different pulse schemes are adopted for depression processes. **a** Identical pulse amplitude modulation: the depression pulse width and interval are set to 50 μs, and the pulse amplitude is fixed to − 5.5 V for all pulses. **b** Nonidentical pulse width modulation: the depression pulse amplitude is fixed to − 5.5V and the interval is set to 50 μs, while the pulse width increases gradually from 2 μs to 256 μs with a step of 2 μs for all 128 erase pulses. **c** Nonidentical pulse amplitude modulation: the depression pulse width and interval are set to 50 μs for all pulses while the pulse amplitude decreases from − 4.53 V to − 5.8V with a step of − 0.01 V. From the results, it is clear that the design of pulse schemes has an obvious modulation effect on the linearity of the depression process.


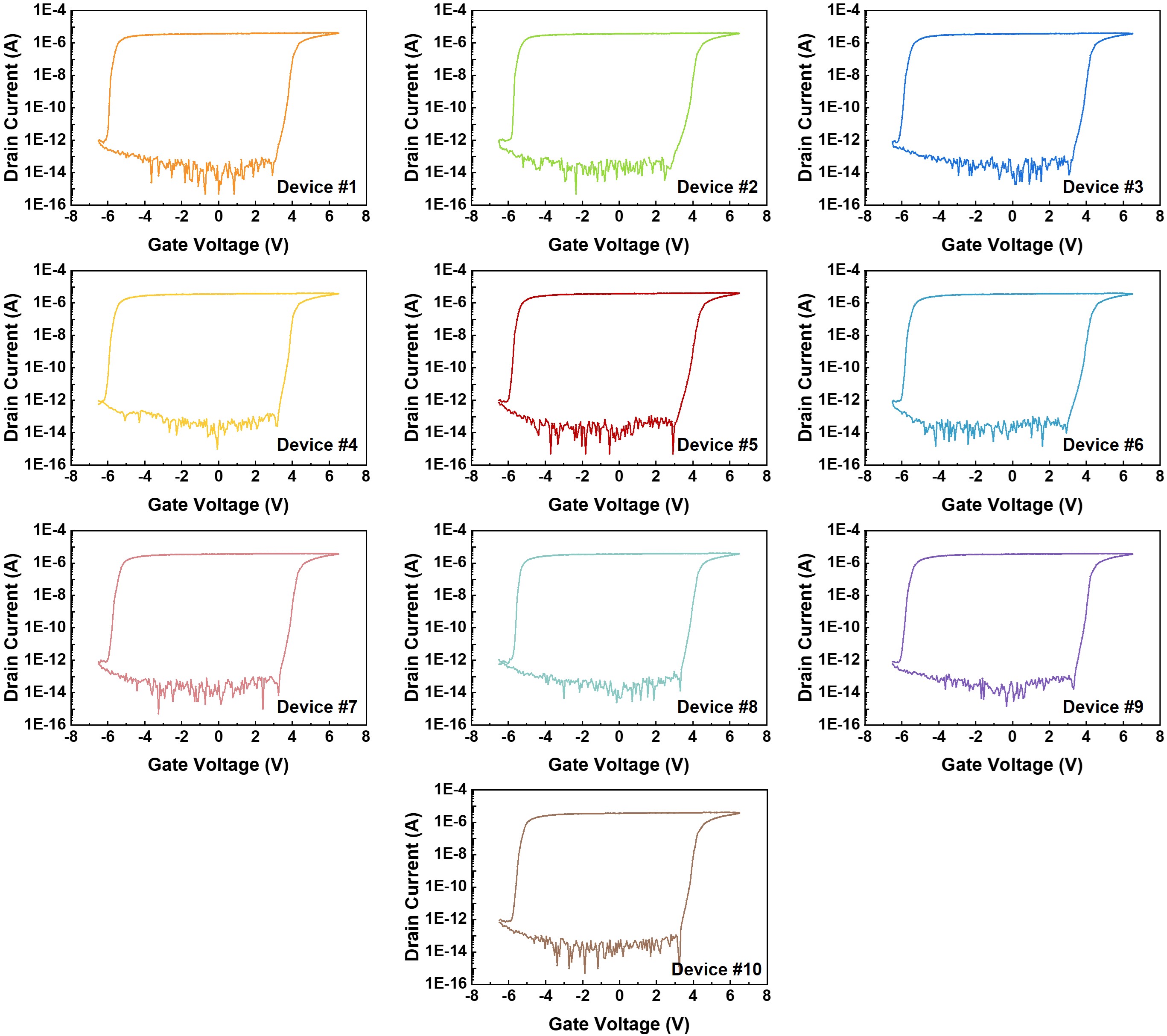


**Fig. S12** Transfer characteristics across ten devices for memory window extraction


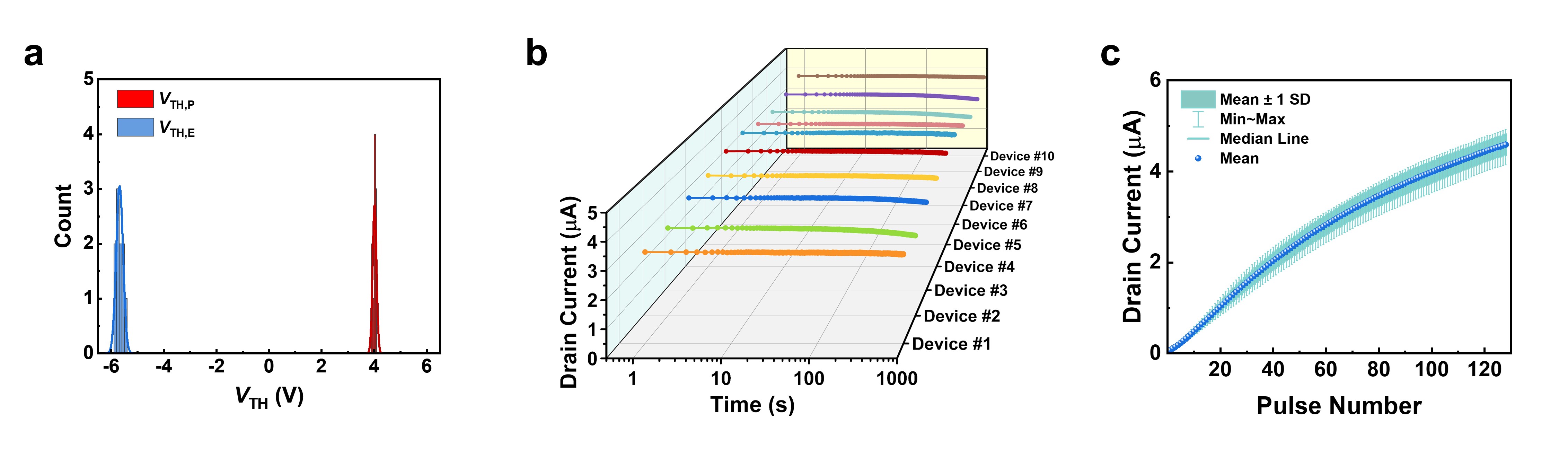


**Fig. S13** Device-to-device variation on nonvolatile behaviors across 10 devices. **a** Distribution of extracted *V*_TH_ for both program and erase states from the measured transfer characteristics of 10 devices. **b** D2D variation in terms of nonvolatile retention. **c** Extraction of D2D variation through long-term potentiation measurement. Ten devices all realize 7-bit synaptic states with a low D2D variation of 9% (the D2D variation is defined as the standard deviation over the mean value σ/μ).

**
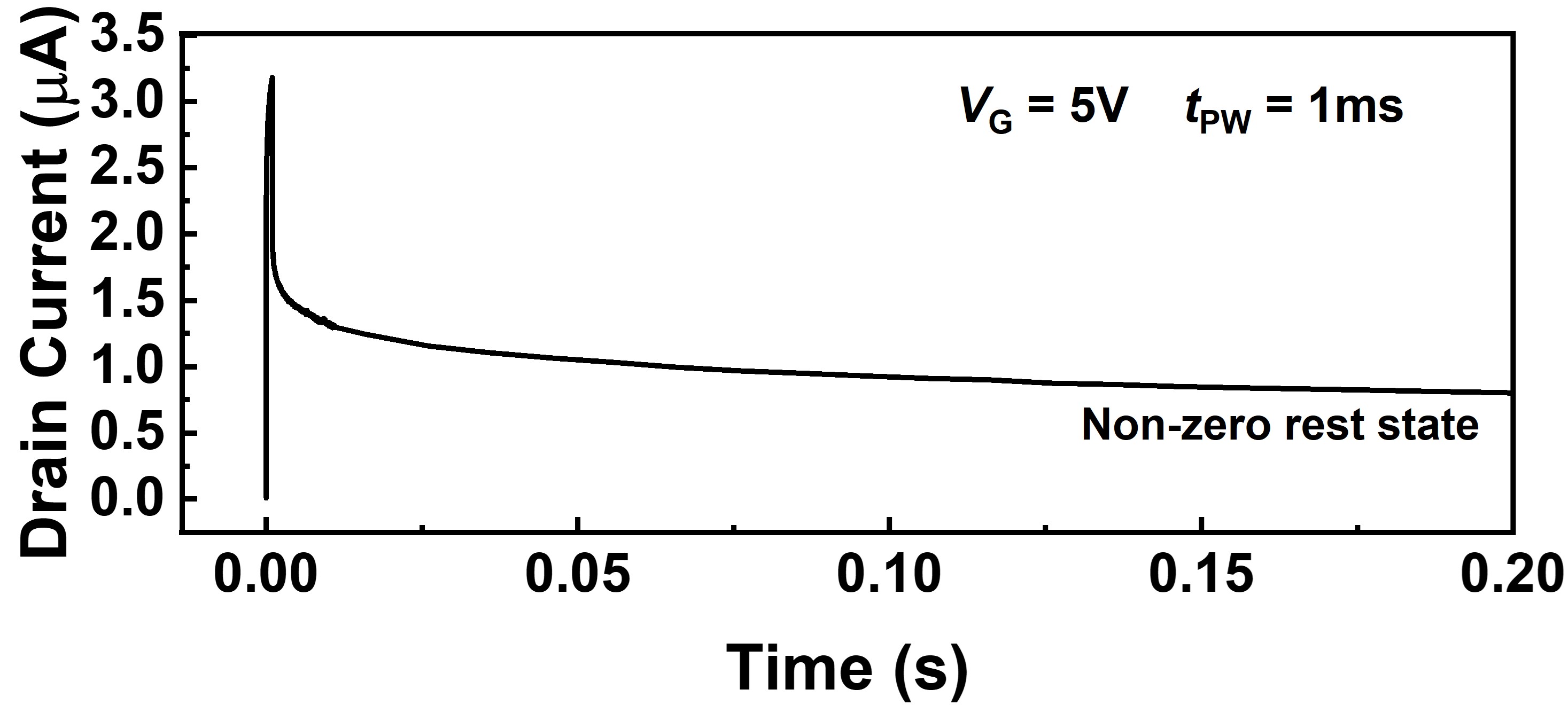
**

**Fig. S14** Recorded drain current response when the FG AFeFET with an AR of 1:5 is stimulated by a single programming gate pulse, clear current integration can be observed within the applied pulse. The obtained drain current decays gradually and finally rests at a non-zero state, which is different from the immediate decay of the drain current back to the initial state at AR = 1:1. The presence of this non-zero state indicates the emergence of the memory effect at this relatively small AR

**Table S3** Benchmarking of coplanar-AFeFET-based ARC system with the conventional reservoir computing systems

| Reference | Device | System Structure | Hardware Cost for Reservoir Layer | Time Constant | Application | Recognition Accuracy |
| --- | --- | --- | --- | --- | --- | --- |
| [S8] | (Pd/Au)/WO_x_/W memristor | Paralleled RC system | 88 memristors | 50 ms | MNIST pattern recognition | 88.1% |
| [S9] | (Pd/Au)/WO_x_/W memristor | Paralleled RC system | 50 memristors | 50ms | Spoken-digit recognition | 99.2% |
| [S10] | (Cr/Au)/SnS/(Cr/Au) memristor | Paralleled RC system | 5 memristors | >10 ms | Korean sentence classification | 91% |
| [S11] | Rotating Neuron Reservoir based on CMOS circuits | Paralleled RC system | 8 parallel cyclic reservoirs | μs–ms regime | MNIST pattern recognition | 94% |
| [S12] | Ferroelectric α-In_2_Se_3_ FESFET | Deep RC system | Multiple transistors | >1s | Waveform Classification | NRMSE = 0.2 |
| This Work | Coplanar FG AFeFET | All-in-one RC with tunable temporal dynamics | Single AFeFET | <1ms | MNIST pattern recognition | 95.6% |
|  |  |  |  |  | Fashion-MNIST pattern recognition | 83.4% |

The conventional RC systems usually adopt two representative structures to expand the reservoir state richness or to realize varying temporal dynamics, including the deep-cascaded RC system structure and the parallel-RC system structure. In deep-cascaded RC systems, multiple physical reservoirs are connected in series, and the input information is sequentially processed by multiple reservoirs. In this way, the reservoir states can only be accessed after the processing of all reservoir layers, resulting in high overall latency and hardware cost growing with the number of layers. Developed from the deep-cascaded RC, the input information is concurrently processed by multiple physical reservoirs in parallel-RC instead of processing sequentially, which greatly improves the processing speed and enhances the system efficiency. However, this kind of system design still requires high hardware costs and large energy consumption as it necessitates multiple devices to increase state diversity and temporal dynamics.

In our coplanar FG AFeFET, controllable temporal dynamics can be generated using a single device by simply modulating the pulsing parameters. In this way, enhanced reservoir state richness and multiple temporal dynamics can be achieved at a low hardware cost. A comparison between the conventional deep-cascaded RC, parallel-RC systems, and the demonstrated AFeFET-based RC is provided in **Table S3**.


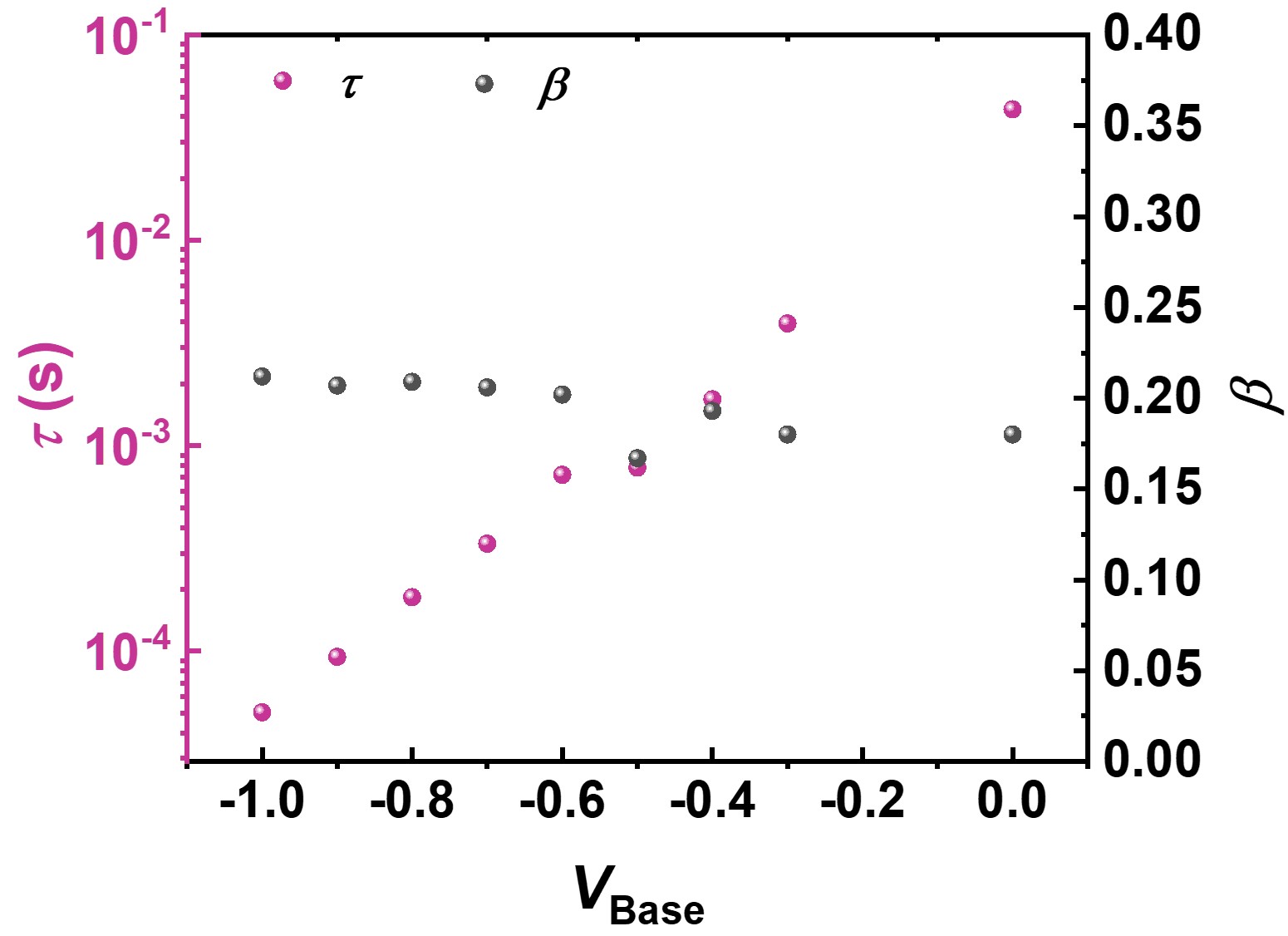


**Fig. S15** The characteristic time constant *τ* and stretch exponent *β* as a function of base voltage *V*_base_, which are extracted from the current decay process using the stretched exponential function. The corresponding measured current decay process under varying *V*_base_ conditions is shown in Fig. 5b. Although the negatively shifted *V*_base_ accelerates the current decay process and generates a smaller time constant *τ*, the stretch exponent *β* only shows negligible variation around a value of 0.2. This almost constant *β* value indicates that the stretched exponential model is suitable for describing the current decay process.


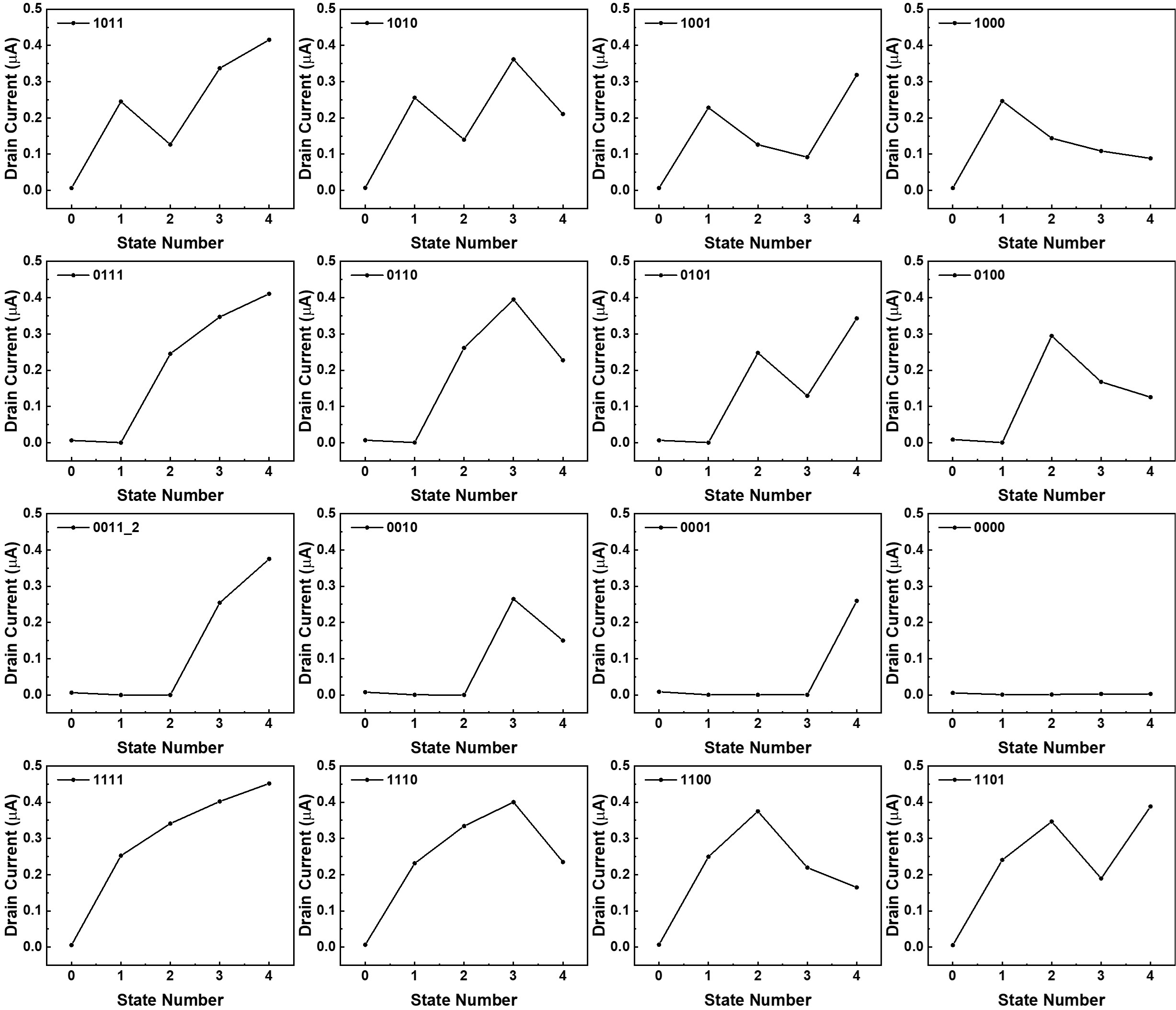


**Fig. S16** Evolution of device response to 16 different combinations of 4-bit input sequences, ranging from (0000) to (1111).

_
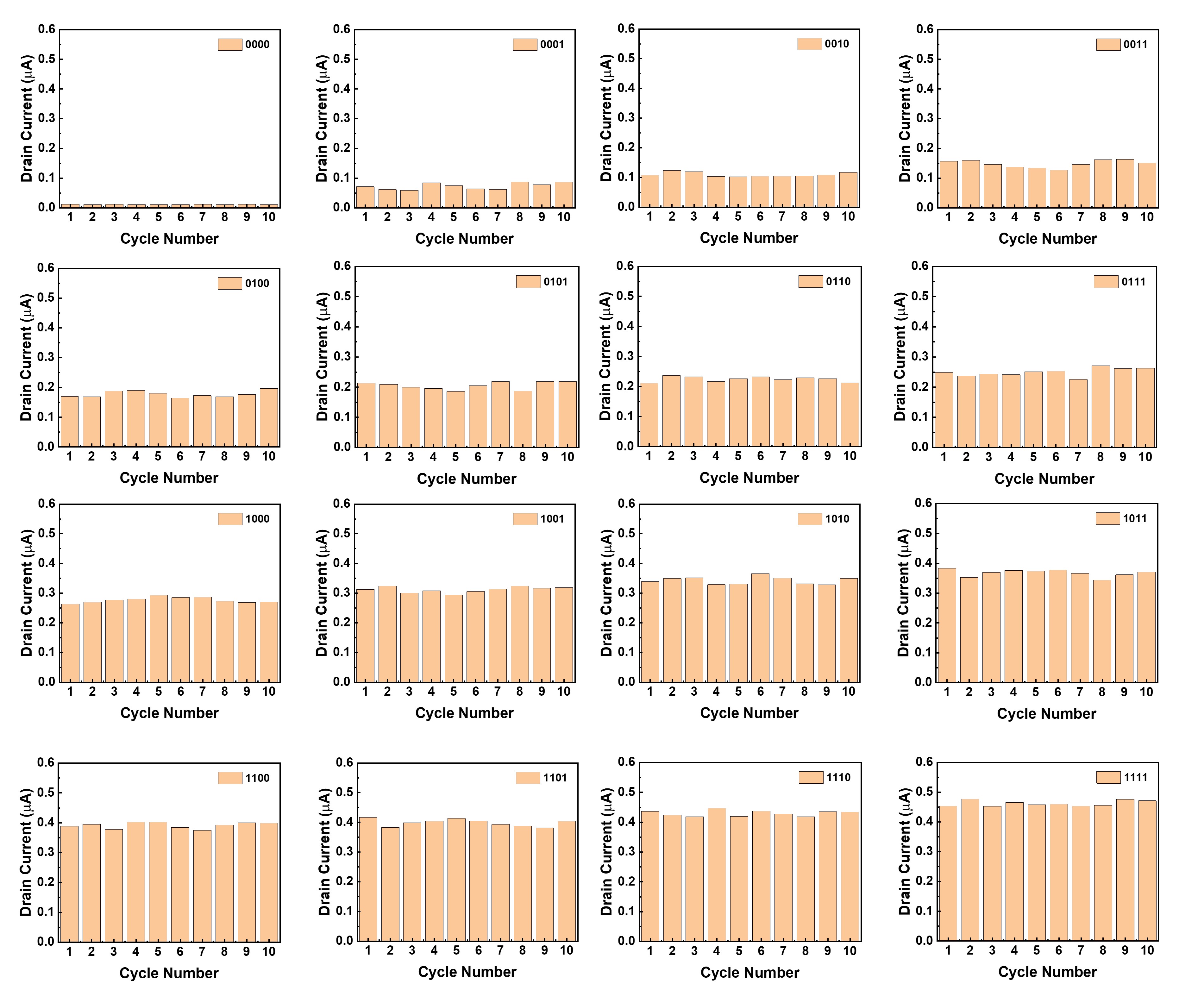
_

**Fig. S17** Evolution of the generated reservoir states of the device in response to 16 different input sequences under 10 repeated cycling tests

_
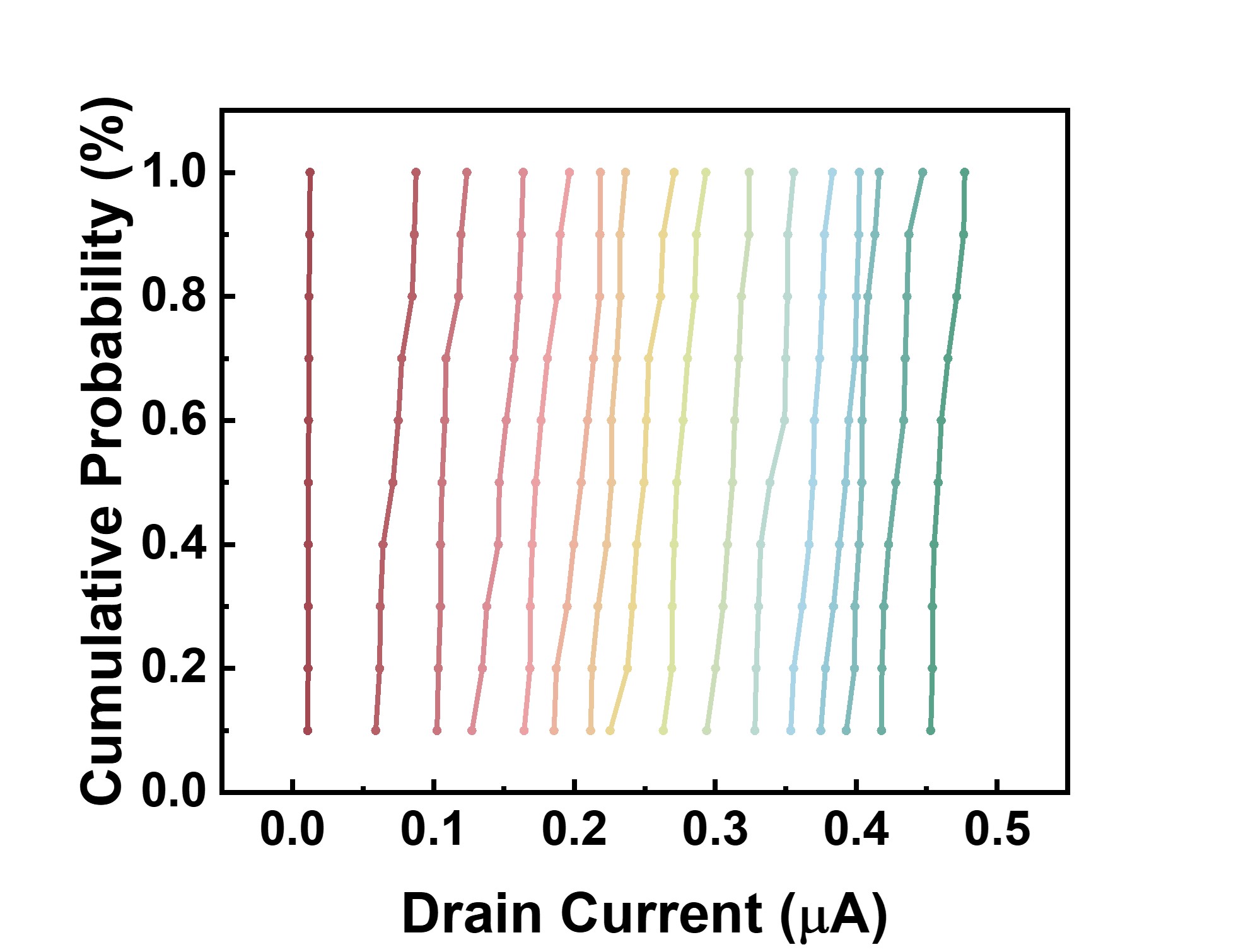
_

**Fig. S18** Cumulative probability distribution of the 16 different reservoir states under 10 measurement cycles. The calculated discrimination between states is calculated as 0.27, indicating a high reservoir state separability of the device

**Table S4.** Benchmarking of coplanar-AFeFET-based ARC system with the state-of-the-art physical reservoirs computing systems. Discrimination between states is defined as the ratio of the standard deviation to the average difference between adjacent reservoir states $D= \sigma/\bar{\Delta}$. The *D* value smaller than 1 refers to a promising state separability

| Device | Number of States | Discrimination between States | Energy Consumption | Time constant | Readout network implementation | Integration of reservoir & readout in a single device | Refs. |
| --- | --- | --- | --- | --- | --- | --- | --- |
| Ti/(TiO_x_/TaO_y_)/Pt  memristor | - | - | 6 nJ | 400 µs | Simulation | No | [S8] |
| (Pd/Au)/WO_x_/W  memristor | - | - | 3 nJ | 50 ms | Simulation | No | [S9] |
| (Cr/Au)/SnS/(Cr/  Au) memristor | 32 | - | ~1.6 nJ | >10 ms | Simulation | No | [S10] |
| Ti/TiO_x_/Pd memristor | - | - | 10 nJ | - | TiN/TaOx/  HfAlOy/TiN memristor | No | [S13] |
| CIPS memristor | - | - | 0.15 nJ | 43.6 ms | Simulation | No | [S14] |
| (Pd/Au)/WO_x_/W  memristor | 16 | 0.79 | 1.5 nJ | 50 ms | Simulation | No | [S15] |
| TiN/NbOₓ/Pt memristor | 16 | - | 7.88 nJ | 20 ms | Simulation | No | [S16] |
| TiN/TaO_x_/HfO_y_/TiN | 16 | - | 0.5 nJ | 28.97 ms | Simulation | No | [S17] |
| IGZO photoelectronic transistor | 16 | - | ≈ 0.26 μJ | ~ 1 s | TaO_X_-based memristor | No | [S18] |
| Pt/BiFeO_3_/SrRuO_3_  Ferroelectric diode | 16 | 1.37 | ~118 nJ | 12 ms | Pt/BiFeO_3_/SrRuO_3_  Ferroelectric diode | Yes | [S19] |
| Mixed-phase boundary thin-film transistor | 16 | 0.61 | 22.5 pJ | 0.25 ms | Mixed-phase boundary thin-film transistor | Yes | [S20] |
| HZO-based coplanar AFeFET | 16 | 0.27 | 24 pJ | 700 μs (tunable:  50 μs – 40 ms) | HZO-based coplanar AFeFET | Yes | This work |

The energy consumption per operation of the coplanar FG AFeFET-based physical reservoir is estimated as follows:

$$E_{reservoir}=I_{D(reservoir)}\times V_{D}\times t_{PW}=240 nA \times0.1 V \times1ms=24 pJ$$

Where *V*_D_ is the read voltage, *I*_D(reservoir)_ is the average current response of the AFeFET-based physical reservoir, and *t*_PW_ is the pulse width. During the calculation, we assume the *I*_D(reservoir)_ of 240 nA from the current evolution of AFeFET across 16 reservoir states (ranging from ~3 nA to ~ 477 nA).


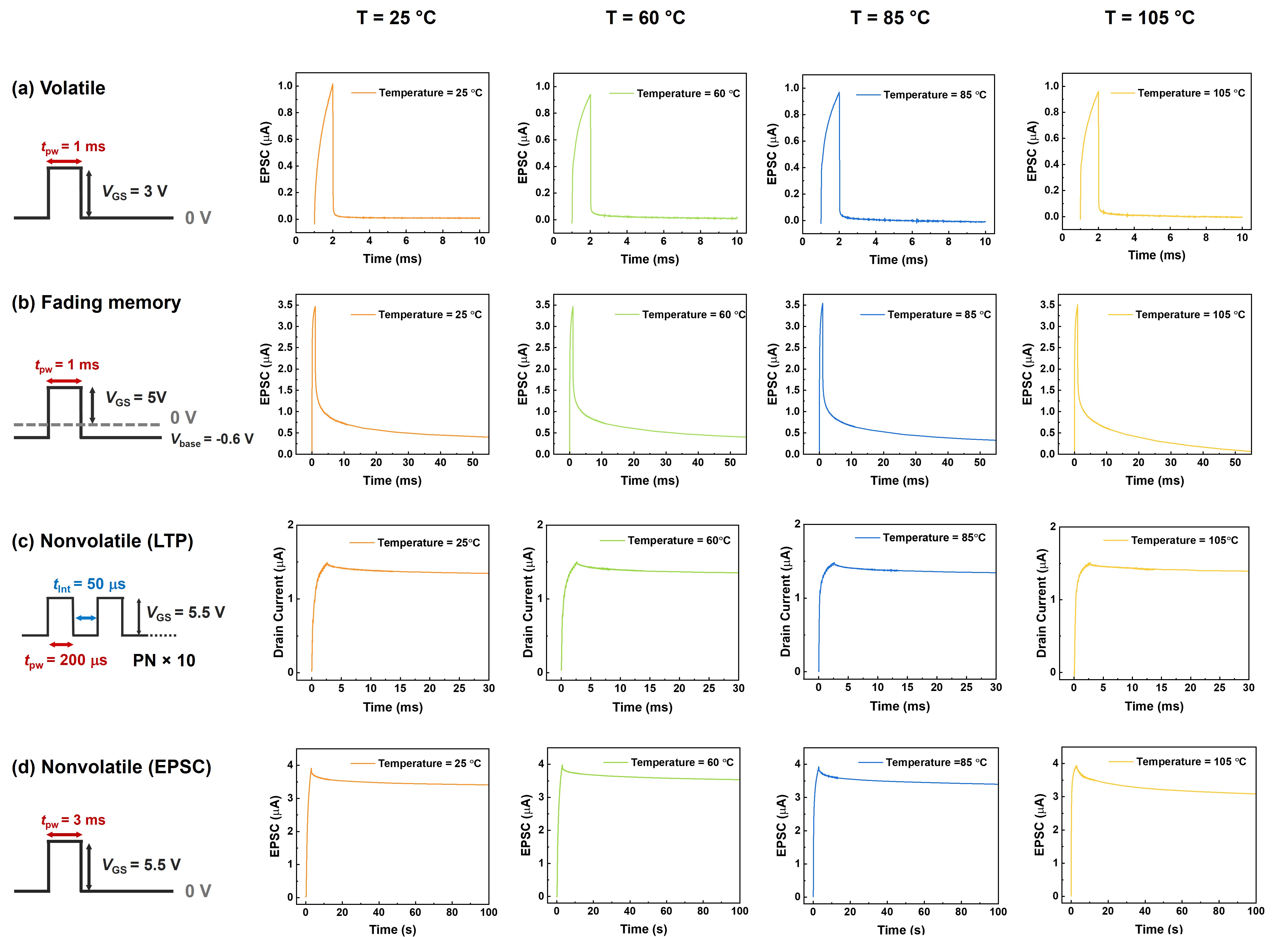


**Fig. S19** Response of coplanar FG AFeFET under varying temperatures across 25°C to 105°C. **a** the volatile behavior obtained through EPSC measurement **b** fading memory behavior obtained through EPSC measurement with non-zero *V*_base_. **c** nonvolatile synaptic response and **d** nonvolatile EPSC measurements

**Note S4 Integration Ability and Application Potential of Coplanar FG AFeFET towards Large-scale Neuromorphic Computing Hardware**

By adopting the proposed coplanar FG structure design, the demonstrated FG AFeFET achieves multiple operation modes within a single device, and a coplanar FG AFeFET-based ARC system is demonstrated as a proof-of-concept. The tunable functionalities of the device make it not only suitable for the physical implementation of ARC systems but also have the potential for demonstrating versatile hardware-based neuromorphic computing systems. Here, the application potential of the device and possible improvements to enhance its integration ability towards large-scale ARC/neuromorphic computing systems are discussed as follows:

1. Integration ability and area efficiency:

By adopting the coplanar structure design, the floating gate layer is formed on the top surface of the device, together with the source and drain formation, which improves vertical scalability by eliminating the need for an internal floating-gate layer in the vertical direction in conventional floating-gate devices. In addition, the required HZO thickness is also reduced to 10 nm compared to previously reported 2D channel-based FG FE/AFeFETs. Nonetheless, the design of three control gates on the same plane may raise concerns regarding the lateral overhead during the implementation of large-scale systems. The following two strategies shown in **Fig. S20** can be adopted to relieve this problem in practical demonstrations, including vertical overlay design and a selective deployment strategy.


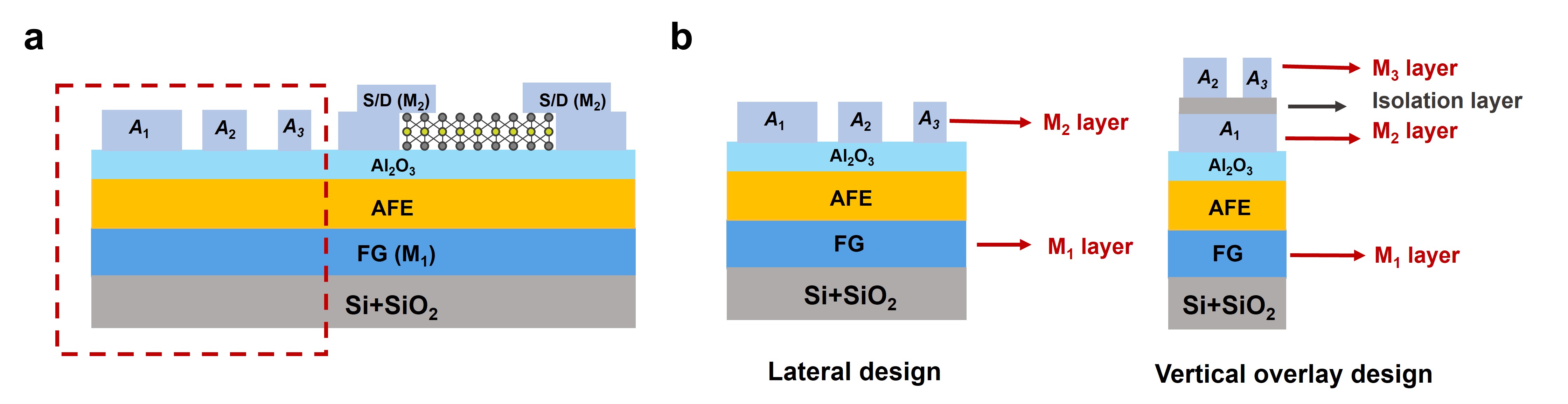


**Fig. S20** **a** Schematic of the coplanar AFeFET with multiple control gates, the red dashed box shows the bottleneck accounts for area overhead in the current design. **b** Schematic of vertical overlay design of the MFIM region. It reduces the area overhead by leveraging the enhanced vertical scalability of the device

1. Vertical overlay design: To reduce the lateral overhead caused by three parallel control gates, and improve the area efficiency, the layout of the MFIM region can be redesigned to stack the control gate 2 (medium AR) and control gate 3 (small AR) on top of control gate 1 (large AR), as shown in **Fig. S20b**. In other words, keep the large-AR control gate in the M2 metal layer, route the medium-AR and small-AR control gates in the M3 metal layer directly above it, and use an inter-metal oxide layer to isolate the M2 and M3 metal layers. In this way, the multiple functionalities are maintained within the device with minimal lateral area consumed.
2. Selective deployment strategy: Among three operation modes that coplanar AFeFET demonstrated, the CG1 responsible for the nonvolatile synapse mode only induces a small *A*_MFIM_ because of its small area ratio, while the control gates responsible for the volatile neuron modes are the main contributing factor to area overhead. Therefore, during the design of a large-scale network, we can design two cell types in the library, including “AFeFET-triple-mode” cell and “AFeFET-mono-mode” cell. In this way, we can utilize “AFeFET-triple-mode” cells in synapse rows and only instantiate “AFeFET-mono-mode” cells at the rows where LIF neuron and graded neuron functions are required.
3. Application potential of coplanar FG AFeFET for versatile neuromorphic computing systems demonstration: The three distinct operating modes of the coplanar FG AFeFET provide great flexibility in designing large-scale neuromorphic computing systems applicable to different scenarios. In this work, a typical analog reservoir computing system is demonstrated as a proof-of-concept to show the capability of the device to function flexibly as different neuromorphic components in different layers based on users’ demand. In this context, in coplanar AFeFET-based neuromorphic systems, the system possesses a post-fabrication flexibility to reassign the role of different devices on demand. Modern edge devices frequently evolve from inference-only (non-volatile synapse layers) to on-device learning or temporal processing (volatile reservoir/LIF neuron layers) during a product’s lifetime. The coplanar AFeFET, with its multiple operation modes on a unified platform, shows high promise for fulfilling this requirement.

**Table S5** Benchmark of key figures of merit of different ferroelectric-material-based devices

| MW/Sweep  Range (V) | On/off ratio | FE layer thickness (nm) | V_P_/V_E_ (V) | Retention (s) | Material | η (%) | Refs. |
| --- | --- | --- | --- | --- | --- | --- | --- |
| 4.7 V/±6 | 10^6^ | 15 | 6/-6 | 10^6^ | HZO/MoS_2_ | 39.2 | [S21] |
| 10 V/±8 | 10^7^ | 18 | 8/-8 | 10^2^ | HZO/WSe_2_ | 62.5 | [S22] |
| 11.5 V/±7.5 | 10^7^ | 20 | 7.5/-7.5 | 4×10^4^ | HZO/MoS_2_ | 76.6 | [S23] |
| 2.5 V/5 | 10^5^ | 6 | 3.5/-3.5 | 2×10^3^ | HZO/WS_2_ | 50 | [S24] |
| 0.22 V/2.25 | 10^4^ | 11 | 3/-2.5 | 5×10^3^ | HZO/MoS_2_ | 10 | [S25] |
| 4 V/±6.5 | N.A. | 6 | 5.5/-5.5 | 10^4^ | HZO/MoS_2_ | 30.7 | [S26] |
| 2 V/6 | 10^6^ | 10 | 5/-1.8 | N.A. | HZO/IGZO | 33.3 | [S27] |
| 8 V/±10 | 10^6^-10^7^ | 45 | 12/-12 | 10^5^ | AlScN/MoS_2_ | 40 | [S28] |
| 23 V/±30 | 7.5×10^3^ | 150 | 20/-20 | 10^3^ | P(VDF-TrFE)  /MoS_2_ | 38.3 | [S29] |
| 11 V/±11 | 10^3^ | 260 | 8/8 | 10^4^ | PZT/MoS_2_ | 50 | [S30] |
| 3.8 V/±4 | 10^7^ | 87 | 5/-5 | 3×10^3^ | CuInP_2_S_6_/MoS_2_ | 47.5 | [S31] |
| 4 V/±3 | 10^4^ | 300 | 3/-3 | 10^3^ | BaSrTiO_3_/MoS_2_ | 67 | [S32] |
| 30 V/±40 | 10^5^ | 100 | 40/-40 | 10^5^ | AlScN/MoS_2_ | 37.5 | [S33] |
| 10 V/±6.5 | **10^7^** | **10** | **5.5/-5.5** | **10^3^** | **HZO/MoS_2_** | **76.9** | **This work** |


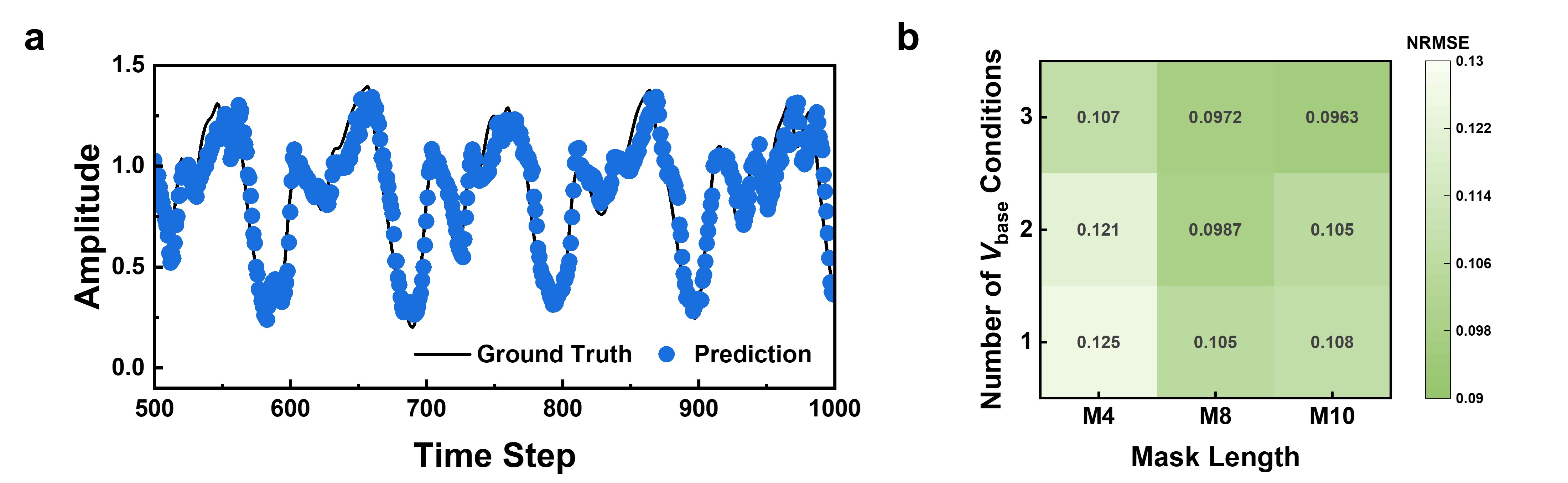


**Fig. S21** **a** The experimentally obtained predicted results and ground truth from the original Mackey–Glass time series. **b** Dependence of NRMSE on the mask length and number of adopted *V*_base_ conditions

To validate the advantages of the coplanar AFeFET-based physical reservoir in processing dynamic and complex tasks, here we perform a time-series forecasting task using the chaotic Mackey-Glass time series that contains temporal and dynamic information. The Mackey-Glass time series has been widely used as a benchmark for forecasting task tests and is generated by a time-delayed differential equation [38] as follows:

$$\frac{dx}{dt}=\beta\frac{x(t-\tau_{MG})}{1+x^{m}(t-\tau_{MG})}-\gamma x\left( t \right) (S7)$$

Where the parameters are set as *β* = 0.2, *γ* = 0.1, *τ*_MG_ = 30, *m* = 10. To achieve accurate predictions, it is important to construct a physical reservoir with high state richness and rich reservoir dynamics to capture the temporal dynamics of the time series. In this context, we leverage the modulation effect of *V*_base_ to create varied reservoir temporal dynamics and subsequently enhance state richness. During the experiment, three *V*_base_ conditions (*V*_base_ = -0.4 V, -0.6 V, -0.8 V) are utilized when the time series is input to generate reservoir states. In addition, a mask with a length of 4 is applied to the input sequence to create virtual nodes and form a delayed feedback system. The prediction result during the test phase is shown in **Fig. S21a**, and the prediction error is evaluated by the calculated normalized root mean square error (NRMSE). The small difference between the prediction and the ground truth, and the obtained low NRMSE of 0.107 indicates the high capability of the physical reservoir in processing dynamic time series.

Besides, the prediction task is further performed under different combinations of the mask length and the number of *V*_base_ conditions. The obtained NRMSE according to different combinations is demonstrated in **Fig. S21b**. Regardless of the adopted mask length, the prediction error is effectively reduced by employing multiple *V*_base_ conditions to leverage the tunable temporal dynamics of the coplanar AFeFET-based reservoir. This result further highlights the advantage of tunable temporal dynamics in improving RC system performance for processing dynamic and complex tasks.

**Supplementary References**

[S1] S.J. Kim, J. Mohan, S.R. Summerfelt, J. Kim, Ferroelectric Hf_0.5_Zr_0.5_O_2_ thin films: A review of recent advances. JOM **71**, 246–255 (2019). <https://doi.org/10.1007/s11837-018-3140-5>

[S2] S.S. Fields, T. Cai, S.T. Jaszewski, A. Salanova, T. Mimura et al., Origin of ferroelectric phase stabilization via the clamping effect in ferroelectric hafnium zirconium oxide thin films. Adv. Electron. Mater. **8**, 2200601 (2022). <https://doi.org/10.1002/aelm.202200601>

[S3] C.-H. Liu, K.-Y. Hsiang, Z.-X. Li, F.-S. Chang, Z.-F. Lou et al., Nonvolatile and Volatile Memory Fusion of Antiferroelectric-like Hafnium–Zirconium Oxide for Multi-Bit Access and Endurance >10^12^ Cycles by Alternating Polarity Cycling Recovery and Spatially Resolved Evolution. ACS Appl. Mater. Interfaces **17**, 14342–14349 (2025). <https://doi.org/10.1021/acsami.4c14132>

[S4] Z. Zheng, L. Jiao, D. Zhang, C. Sun, Z. Zhou et al., BEOL-compatible MFMIS ferroelectric/anti-ferroelectric FETs—Part I: experimental results with boosted memory window. IEEE Trans. Electron Devices **71**, 1–7 (2023). <https://doi.org/10.1109/TED.2023.3326116>

[S5] Z. Zheng, D. Zhang, L. Jiao, C. Sun, Z. Zhou et al., BEOL-compatible MFMIS ferroelectric/ anti-ferroelectric fets—part ii: mechanism with load line analysis and scaling strategy. IEEE Trans. Electron Devices **71**, 5325–5331 (2024). <https://doi.org/10.1109/TED.2024.3421184>

[S6] W. Wei, W. Zhang, L. Tai, G. Zhao, P. Sang et al., In-depth understanding of polarization switching kinetics in polycrystalline Hf_0.5_Zr_0.5_O_2_ ferroelectric thin film: a transition from NLS to KAI. in 2021 IEEE International Electron Devices Meeting (IEDM), (IEEE, San Francisco, CA, USA, 2021)., pp. 19.1.1-19.1.4

[S7] J. Y. Jo, H. S. Han, J.-G. Yoon, T. K. Song, S.-H. Kim et al., Domain switching kinetics in disordered ferroelectric thin films. Phys. Rev. Lett. **99**, 267602 (2007). <https://doi.org/10.1103/PhysRevLett.99.267602>

[S8] Y. Zhong, J. Tang, X. Li, B. Gao, H. Qian et al., Dynamic memristor-based reservoir computing for high-efficiency temporal signal processing. Nat. Commun. **12**, 408 (2021). <https://doi.org/10.1038/s41467-020-20692-1>

[S9] J. Moon, W. Ma, J.H. Shin, F. Cai, C. Du et al., Temporal data classification and forecasting using a memristor-based reservoir computing system. Nat. Electron. **2**, 480–487 (2019). <https://doi.org/10.1038/s41928-019-0313-3>

[S10] L. Sun, Z. Wang, J. Jiang, Y. Kim, B. Joo et al., In-sensor reservoir computing for language learning via two-dimensional memristors. Sci. Adv. **7**, eabg1455 (2021). <https://doi.org/10.1126/sciadv.abg1455>

[S11] X. Liang, Y. Zhong, J. Tang, Z. Liu, P. Yao et al., Rotating neurons for all-analog implementation of cyclic reservoir computing. Nat. Commun. **13**, 1549 (2022). <https://doi.org/10.1038/s41467-022-29260-1>

[S12] K. Liu, B. Dang, T. Zhang, Z. Yang, L. Bao et al., Multilayer Reservoir Computing Based on Ferroelectric α‐In_2_Se_3_ for Hierarchical Information Processing. Adv. Mater. **34**, 2108826 (2022). <https://doi.org/10.1002/adma.202108826>

[S13] Y. Zhong, J. Tang, X. Li, X. Liang, Z. Liu et al., A memristor-based analogue reservoir computing system for real-time and power-efficient signal processing. Nat. Electron. **5**, 672–681 (2022). <https://doi.org/10.1038/s41928-022-00838-3>

[S14] Y. Wu, N. T. Duong, Y. Chien, S. Liu, K. Ang, A Dynamic Memory for Reservoir Computing Utilizing Ion Migration in CuInP_2_S_6_. Adv. Electron. Mater. **10**, 2300481 (2024). <https://doi.org/10.1002/aelm.202300481>

[S15] C. Du, F. Cai, M. A. Zidan, W. Ma, S. H. Lee et al., Reservoir computing using dynamic memristors for temporal information processing. Nat. Commun. **8**, 2204 (2017). <https://doi.org/10.1038/s41467-017-02337-y>

[S16] S. Hua, L. Zhang, L. Wang, R. Zheng, P. Gan et al., Electrochemical Preparation of Reliable and High Yield Memristors for Efficient Reservoir Computing Systems. ACS Appl. Mater. Interfaces **17**, 53691–53703 (2025). <https://doi.org/10.1021/acsami.5c10190>

[S17] Z. Zhang, X. Zhao, X. Zhang, X. Hou, X. Ma et al., In-sensor reservoir computing system for latent fingerprint recognition with deep ultraviolet photo-synapses and memristor array. Nat. Commun. **13**, 6590 (2022). <https://doi.org/10.1038/s41467-022-34230-8>

[S18] H. Cui, Y. Xiao, Y. Yang, M. Pei, S. Ke et al., A bioinspired in-materia analog photoelectronic reservoir computing for human action processing. Nat. Commun. **16**, 2263 (2025). <https://doi.org/10.1038/s41467-025-56899-3>

[S19] Z. Chen, W. Li, Z. Fan, S. Dong, Y. Chen et al., All-ferroelectric implementation of reservoir computing. Nat. Commun. **14**, 3585 (2023). <https://doi.org/10.1038/s41467-023-39371-y>

[S20] J. Kim, E. C. Park, W. Shin, R.-H. Koo, C.-H. Han et al., Analog reservoir computing via ferroelectric mixed phase boundary transistors. Nat. Commun. **15**, 9147 (2024). <https://doi.org/10.1038/s41467-024-53321-2>

[S21] T. Lu, J. Xue, P. Shen, H. Liu, X. Gao et al., Two-dimensional fully ferroelectric-gated hybrid computing-in-memory hardware for high-precision and energy-efficient dynamic tracking. Sci. Adv. **10**, eadp0174 (2024). <https://doi.org/10.1126/sciadv.adp0174>

[S22] J. Huo, L. Li, H. Zheng, J. Gao, T.T.T. Tun et al., Compact physical implementation of spiking neural network using ambipolar WSe_2_ n-Type/p-Type ferroelectric field-effect transistor. ACS Nano **18**, 28394–28405 (2024). <https://doi.org/10.1021/acsnano.4c11081>

[S23] H. Xiang, Y. Chien, L. Li, H. Zheng, S. Li et al., Enhancing memory window efficiency of ferroelectric transistor for neuromorphic computing via two‐dimensional materials integration. Adv. Funct. Mater. **33**, 2304657 (2023). <https://doi.org/10.1002/adfm.202304657>

[S24] L. Chen, L. Wang, Y. Peng, X. Feng, S. Sarkar et al., A van der Waals synaptic transistor ased on ferroelectric Hf_0.5_Zr_0.5_O_2_ and 2D tungsten disulfide. Adv. Elect. Mater. **6**, 2000057 (2020). <https://doi.org/10.1002/aelm.202000057>

[S25] J. Xiang, W. H. Chang, T. Saraya, T. Hiramoto, T. Irisawa et al., Experimental demonstration of HfO_2_-based ferroelectric FET with MoS_2_ channel for high-density and low-power memory application. in 2021 Silicon Nanoelectronics Workshop (SNW), (2021)., pp. 1–2

[S26] S. Zhang, Y. Liu, J. Zhou, M. Ma, A. Gao et al., Low voltage operating 2D MoS_2_ ferroelectric memory transistor with Hf_1-x_Zr_x_O_2_ gate structure. Nanoscale Res. Lett. **15**, 157 (2020). <https://doi.org/10.1186/s11671-020-03384-z>

[S27] S.-H. Tsai, Z. Fang, X. Wang, U. Chand, C.-K. Chen et al., Stress-memorized HZO for high-performance ferroelectric field-effect memtransistor. ACS Appl. Electron. Mater. **4**, 1642–1650 (2022). <https://doi.org/10.1021/acsaelm.1c01321>

[S28] K.-H. Kim, S. Oh, M.M.A. Fiagbenu, J. Zheng, P. Musavigharavi et al., Scalable CMOS back-end-of-line-compatible AlScN/two-dimensional channel ferroelectric field-effect transistors. Nat. Nanotechnol. **18**, 1044–1050 (2023). <https://doi.org/10.1038/s41565-023-01399-y>

[S29] Z.-D. Luo, S. Zhang, Y. Liu, D. Zhang, X. Gan et al., Dual-ferroelectric-coupling-engineered two-dimensional transistors for multifunctional in-memory computing. ACS Nano **16**, 3362–3372 (2022). <https://doi.org/10.1021/acsnano.2c00079>

[S30] Xiao-Wen Zhang, Dan Xie, Jian-Long Xu, Yi-Lin Sun, Xian Li et al., MoS_2_ field-effect transistors with lead zirconate-titanate ferroelectric gating. IEEE Electron Device Lett. **36**, 784–786 (2015). <https://doi.org/10.1109/LED.2015.2440249>

[S31] X. Wang, C. Zhu, Y. Deng, R. Duan, J. Chen et al., Van der Waals engineering of ferroelectric heterostructures for long-retention memory. Nat. Commun. **12**, 1109 (2021). <https://doi.org/10.1038/s41467-021-21320-2>

[S32] Y. Jeong, H.-J. Jin, J. H. Park, Y. Cho, M. Kim et al., Low voltage and ferroelectric 2D electron devices using lead-free Ba_x_Sr_1-x_TiO_3_ and MoS_2_ channel. Adv. Funct. Mater. **30**, 1908210 (2020). <https://doi.org/10.1002/adfm.201908210>

[S33] X. Liu, D. Wang, K.-H. Kim, K. Katti, J. Zheng et al., Post-CMOS compatible aluminum scandium nitride/2d channel ferroelectric field-effect-transistor memory. Nano Lett. **21**, 3753–3761 (2021). <https://doi.org/10.1021/acs.nanolett.0c05051>
